# Conditional ablation of DIS3L2 ribonuclease in pre-meiotic germ cells causes defective spermatogenesis and infertility in male mice

**DOI:** 10.7150/thno.98620

**Published:** 2024-09-03

**Authors:** Nana Li, Junjie Yu, Yan-Qin Feng, Phoebe Xu, Xiao Wang, Meiyang Zhou, Hong Li, Yu Xu, Zhengpin Wang

**Affiliations:** 1 Shandong Provincial Key Laboratory of Animal Cell and Developmental Biology, School of Life Sciences, Shandong University, Qingdao 266237, China.; 2 Enloe High School, Raleigh, North Carolina 27610, USA.

**Keywords:** spermatogonia, meiosis, spermatogenesis, infertility, scRNA-seq

## Abstract

**Rationale:** Spermatogenesis is a highly organized cell differentiation process in mammals, involving mitosis, meiosis, and spermiogenesis. DIS3L2, which is primarily expressed in the cytoplasm, is an RNA exosome-independent ribonuclease. In female mice, *Dis3l2*-deficient oocytes fail to resume meiosis, resulting in arrest at the germinal vesicle stage and complete infertility. However, the role of DIS3L2 in germ cell development in males has remained largely unexplored.

**Methods:** We established a pre-meiotic germ cell conditional knockout mouse model and investigated the biological function of DIS3L2 in spermatogenesis and male fertility through bulk RNA-seq and scRNA-seq analyses.

**Results:** This study unveils that conditional ablation of *Dis3l2* in pre-meiotic germ cells with *Stra8-Cre* mice impairs spermatogonial differentiation and hinders spermatocyte meiotic progression coupled with cell apoptosis. Such conditional ablation leads to defective spermatogenesis and sterility in adults. Bulk RNA-seq analysis revealed that *Dis3l2* deficiency significantly disrupted the transcriptional expression pattern of genes related to the cell cycle, spermatogonial differentiation, and meiosis in *Dis3l2* conditional knockout testes. Additionally, scRNA-seq analysis indicated that absence of DIS3L2 in pre-meiotic germ cells causes disrupted RNA metabolism, downregulated expression of cell cycle genes, and aberrant expression of spermatogonial differentiation genes, impeding spermatogonial differentiation. In meiotic spermatocytes, loss of DIS3L2 results in disturbed RNA metabolism, abnormal translation, and disrupted meiotic genes that perturb meiotic progression and induce cell apoptosis, leading to subsequent failure of spermatogenesis and male infertility.

**Conclusions:** Collectively, these findings highlight the critical role of DIS3L2 ribonuclease-mediated RNA degradation in safeguarding the correct transcriptome during spermatogonial differentiation and spermatocyte meiotic progression, thus ensuring normal spermatogenesis and male fertility.

## Introduction

Spermatogenesis is a complex process of cell differentiation that results in the production of mature spermatozoa, sustaining male fertility throughout adulthood. It consists of three distinct phases: mitosis, meiosis, and spermiogenesis 1, 2. Spermatogonial stem cells undergo amplification, generating a population of undifferentiated spermatogonia, which further differentiate into differentiated spermatogonia. Type B spermatogonia give rise to two primary spermatocytes. These cells undergo processes including DNA replication, synapsis, and meiotic recombination and ultimately divide twice to produce haploid round spermatids, initiating spermiogenesis and forming mature spermatozoa 3.

Considerable investigations have focused on discovering developmentally regulated transcriptional networks required for mouse spermatogenesis 4-7. Multiple RNases and co-factors are involved in developmentally the correct transcriptome to ensure normal spermatogenesis in mice 8-11. For example, the catalytic ribonuclease EXOSC10 (yeast homolog, RRP6) mediates RNA decay in the nucleus and nucleolus 12, 13. EXOSC10 is associated with epigenetic chromosome silencing and is essential for male germ cell proliferation and development 8. During the early leptotene and zygotene stages of male germ cell meiosis, transcription remains inactive when synapsis occurs 14. Transcription resumes at the pachytene stage, coinciding with meiotic recombination 15-17. A recent study has reported that the zygotene to pachytene transition is not only associated with transcription resumption but also with a programmed wave of mRNA degradation, which is essential for meiotic progression 9. Thus, investigations of the transcriptome networks provide insights into the underlying molecular mechanisms that support spermatogenesis and ensue male fertility in mammals.

DIS3-like exonuclease 2 (DIS3L2), a homolog of DIS3, is an RNA-exosome independent ribonuclease primarily expressed in the cytoplasm 18, 19. Germline mutations in *DIS3L2* have been reported to lead to Perlman syndrome, characterized by overgrowth, and increased susceptibility to Wilms tumor 19. Recently, it has been demonstrated that DIS3L2-mediated decay safeguards endoplasmic reticulum-targeted mRNA translation and maintains calcium ion homeostasis 20. Additionally, DIS3L2 has been shown to regulate cell proliferation and tissue growth in both humans and flies 21. Furthermore, recent studies have delved into the role of DIS3L2 in female reproduction, revealing that *Dis3l2*-deficient oocytes nearly arrest at the germinal vesicle stage, resulting in total infertility in female mice 22. Upon DIS3L2 depletion, uridylated-poly(A) RNAs remain intact and become prevalent in the transcriptome of *Dis3l2*-deficient oocytes. These uridylated-poly(A) RNAs possess shorter poly(A) tails, significantly reducing their translational activity 22. Despite the emerging understanding of DIS3L2's functions in female reproduction and associated human diseases, its significance in male reproduction remains unclear.

In this study, germ cell *Dis3l2* conditional knockout (cKO) mice were established, and the results revealed that the loss of DIS3L2 severely impairs spermatogonial differentiation and hampers spermatocyte meiotic progression. This results in notable defects in spermatogenesis and, ultimately, leads to male sterility. Comparative RNA-seq and scRNA-seq analyses revealed severe dysregulation of the transcriptome during spermatogonial differentiation and spermatocyte meiosis in *Dis3l2* cKO testes. Altogether, the data of this study strongly suggest that DIS3L2 plays a pivotal role in sculpting the transcriptome during spermatogonial differentiation and spermatocyte meiotic progression, ensuring normal spermatogenesis and male fertility.

## Results

### *Dis3l2* expression in the mouse testes

It has previously been reported that DIS3L2 functions as an RNA-exosome independent ribonuclease and is primarily expressed in the cytoplasm. To investigate the role of DIS3L2 in mouse testes, *Dis3l2* transcript levels were initially assessed using recently published scRNA-seq data from adult mouse testes 4. *Dis3l2* transcripts were detected in various cell types within the adult mouse testes, including spermatogonia, spermatocytes, spermatids, Sertoli cells, and Leydig cells (Figure S1A, B). To further delineate the expression pattern of *Dis3l2* transcripts in testicular cell types, given the absence of commercially available antibodies for immunofluorescence, an *in situ* hybridization mRNA analysis of *Dis3l2* was conducted in the testes of P8, P12, and P35 wild-type mice. These analyses conclusively demonstrated that *Dis3l2* was indeed expressed in the spermatogonia, spermatocytes, and round spermatids (Figure S1C). These findings indicate the presence of *Dis3l2* in pre-meiotic germ cells and spermatocytes, thus providing the foundation for a conditional ablation of *Dis3l2* in the testes to elucidate the function of DIS3L2 in mouse spermatogenesis.

### DIS3L2 is essential for spermatogenesis and male fertility

**Figure 1:**
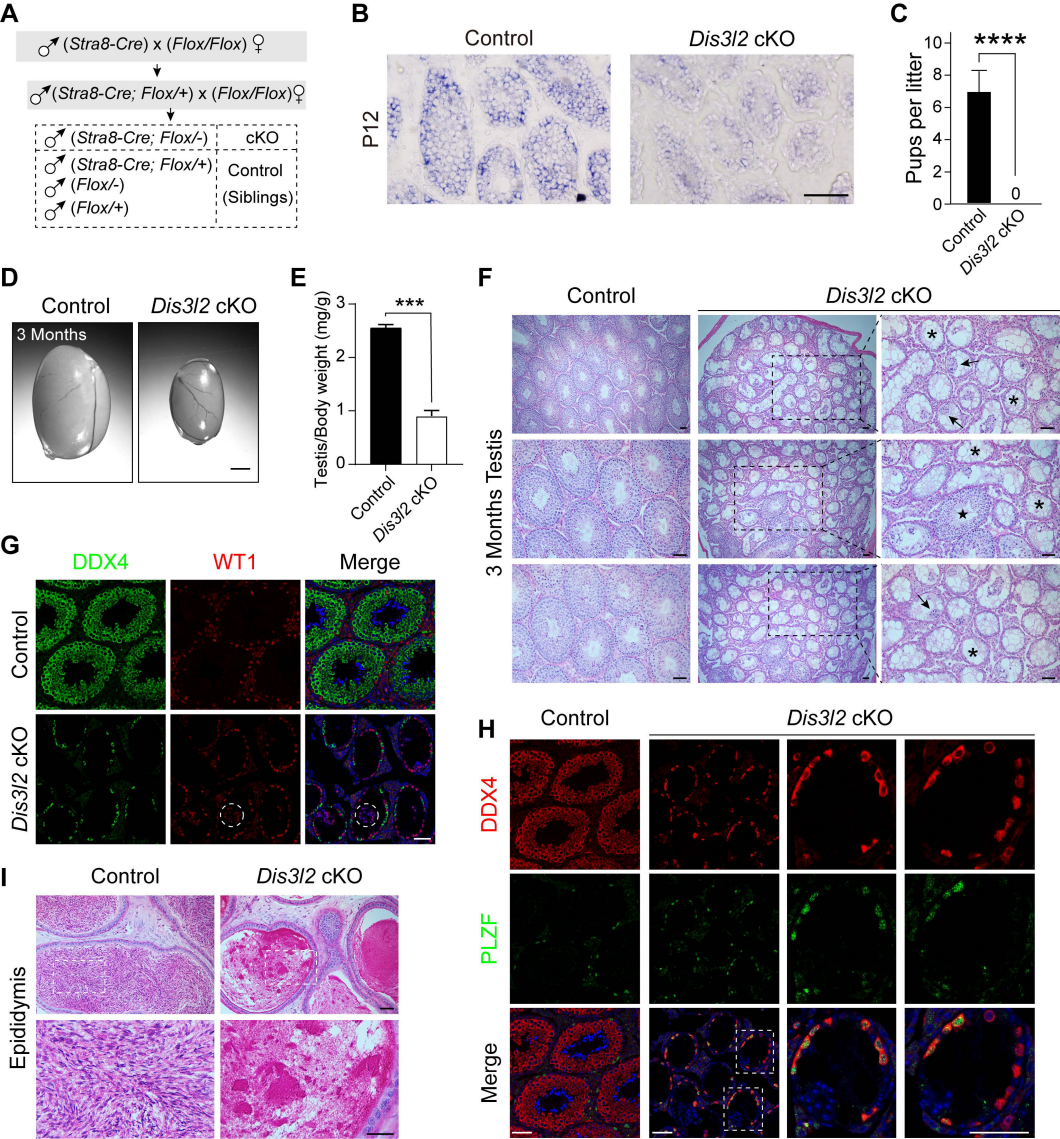
** DIS3L2 is required for spermatogenesis and male fertility.** (A) Schematic diagram showing the establishment of the *Dis3l2* cKO male mouse model. (B) *In situ* hybridization mRNA analysis of *Dis3l2* in P12 control and *Dis3l2* cKO mouse testes. Scale bar, 50 μm. (C) Fertility test showing the sterility of *Dis3l2* cKO male mice. Mean number of litters ± s.d. ***P < 0.001. (D, E) Morphology of testes (D) and the ratio of testis to body weight (E) in control and *Dis3l2* cKO mice at 3 months. Scale bar, 1 mm. Mean ± s.d., n = 4 biologically independent testes from four different animals. ***P < 0.001. (F) Testicular sections of control and *Dis3l2* cKO mice collected at 3 months and stained with periodic acid-Schiff and hematoxylin. Asterisks indicate Sertoli cell-only tubules, star indicates normal seminiferous tubule, and arrows indicate multinucleated giant Sertoli cells. Scale bar, 50 μm. (G) Immunofluorescence staining of testis tissue sections of control and *Dis3l2* cKO mice at 3 months after staining with antibodies against DDX4 (left) or WT1 (middle), followed by staining with Hoechst 33342 to detect DNA (right). Scale bar, 50 μm. Dashed circles indicate multinucleated giant Sertoli cells. (H) Dual-immunofluorescence staining with DDX4 and PLZF antibodies in the testis tissue of control and *Dis3l2* cKO mice at 3 months. Dashed rectangles indicate enlarged areas. Scale bar, 50 μm. (I) Cauda epididymides of control and *Dis3l2* cKO mice at 3 months and stained with periodic acid-Schiff and hematoxylin. Scale bar, 50 μm. Representative of n = 3 (B, F-I) independent biological replicates with similar results per condition.

To investigate the role of DIS3L2 in spermatogenesis, *Dis3l2* was conditionally disrupted by crossing *Dis3l2^Floxed/Floxed^ (Dis3l2^F/F^*) mice (Figure S2A) with *Stra8-Cre* mice (Figure 1A). In these mice, CRE recombinase is initially expressed at P3, affecting part of spermatogonia through all pre-leptotene stage spermatocytes 23. *Stra8-Cre*; *Dis3l2^F/-^* mice (referred to as *Dis3l2* cKO) and their littermate controls were obtained and confirmed through genomic PCR assay (Figure S2B). *In situ* hybridization mRNA analysis of* Dis3l2* in P12 control and *Dis3l2* cKO mouse testes revealed the absence of *Dis3l2* in pre-meiotic germ cells and spermatocytes (Figure 1B). Moreover, quantitative RT-PCR assay revealed a significant reduction in the *Dis3l2* mRNA expression level in P12 *Dis3l2* cKO testes compared with those of controls (Figure S1D). In search of a commercially available antibody of DIS3L2, one antibody was finally identified and was available for immunoblot. Immunoblot assay documented a significant reduction in DIS3L2 protein abundance in the P12 *Dis3l2* cKO testes compared with control testes (Figure S1E). These results indicate the successful establishment of a conditional KO mouse line with specific ablation of *Dis3l2* in pre-meiotic germ cells and meiotic spermatocytes. Subsequently, the reproductive phenotypes of *Dis3l2* cKO male mice were investigated. Over a 6-month mating period, *Dis3l2* cKO males proved to be completely sterile (Figure 1C) and exhibited significantly smaller testes with a reduced ratio of testis to body weight at 3 months (Figure 1D, E). The histological analysis documented a range of anomalies in *Dis3l2* cKO testes (Figure 1F). The great majority of the seminiferous tubules were devoid of germ cells and lined only with Sertoli cells (asterisks), some tubules featured multinucleated giant Sertoli cells (arrows), a few tubules exhibited reduced numbers of spermatogenic cells and vacuoles, and very few tubules displayed normal spermatogenesis with all stages of spermatogenic cells (star). To further validate the observed defects in spermatogenesis in *Dis3l2* cKO testes as shown by PAS staining, the dual-immunofluorescence staining of DEAD-box helicase 4 (DDX4, a germ cell-specific marker) and Wilms tumor 1 (WT1, a Sertoli cell-specific marker) was performed (Figure 1G). The co-immunostaining results revealed that only a single layer of DDX4-positive cells was present at the basement membrane of the epithelium, and WT1-positive cells were fused, forming multinucleated giant Sertoli cells (dashed circles) in some mutant tubules, consistent with observations in testicular sections after PAS staining. In *Dis3l2* cKO testes with only Sertoli cells, a single layer of germ cells was observed at the epithelium (Figure 1G), indicating disruption in spermatogenic cell differentiation. To identify these germ cells at the epithelium, DDX4 and promyelocytic leukemia zinc finger, PLZF (official name ZBTB16, a marker for undifferentiated spermatogonia) 24, were co-stained (Figure 1H). The results indicated that nearly all DDX4-positive cells were also PLZF-positive, suggesting they were undifferentiated spermatogonia. Co-immunostaining of PLZF and Cyclin D1 (a marker for mitotically active spermatogonia) demonstrated that these PLZF-positive spermatogonia displayed high mitotic activity in adult *Dis3l2* cKO testes (Figure S3A), implying normal maintenance of undifferentiated spermatogonia. As *Stra8-Cre*-mediated recombination starts from type A_1_ spermatogonia, co-expression of GDNF family receptor alpha 1 (GFRA1) and Cyclin D1 indicated that GFRA1-positive undifferentiated spermatogonia (potential spermatogonial stem cells) were mitotically active (Figure S3B), revealing normal self-renewal and proliferation of spermatogonial stem cells in adult *Dis3l2* cKO testes. Immunofluorescence staining with antibodies to DDX4 and stem cell growth factor receptor (KIT, a marker for differentiated spermatogonia) documented that KIT-positive spermatogonia were barely detected in adult *Dis3l2* cKO testes, whereas a population of KIT-positive spermatogonia was present in adult control testes (Figure S3C), suggesting compromised spermatogonial differentiation in adult *Dis3l2* cKO testes. Collectively, these results imply a significant compromise in spermatogonial differentiation and spermatogenic lineage development in adult *Dis3l2* cKO testes. Consistently, almost no spermatozoa were present in the cauda epididymis of *Dis3l2* cKO mice (Figure 1I). Taken together, these findings underscore the essential role of DIS3L2 in mouse spermatogenesis, with its ablation resulting in dramatic loss of spermatogenic cells and subsequent male infertility.

### *Dis3l2* deletion causes severe spermatogenic cell loss during the first wave of spermatogenesis

**Figure 2:**
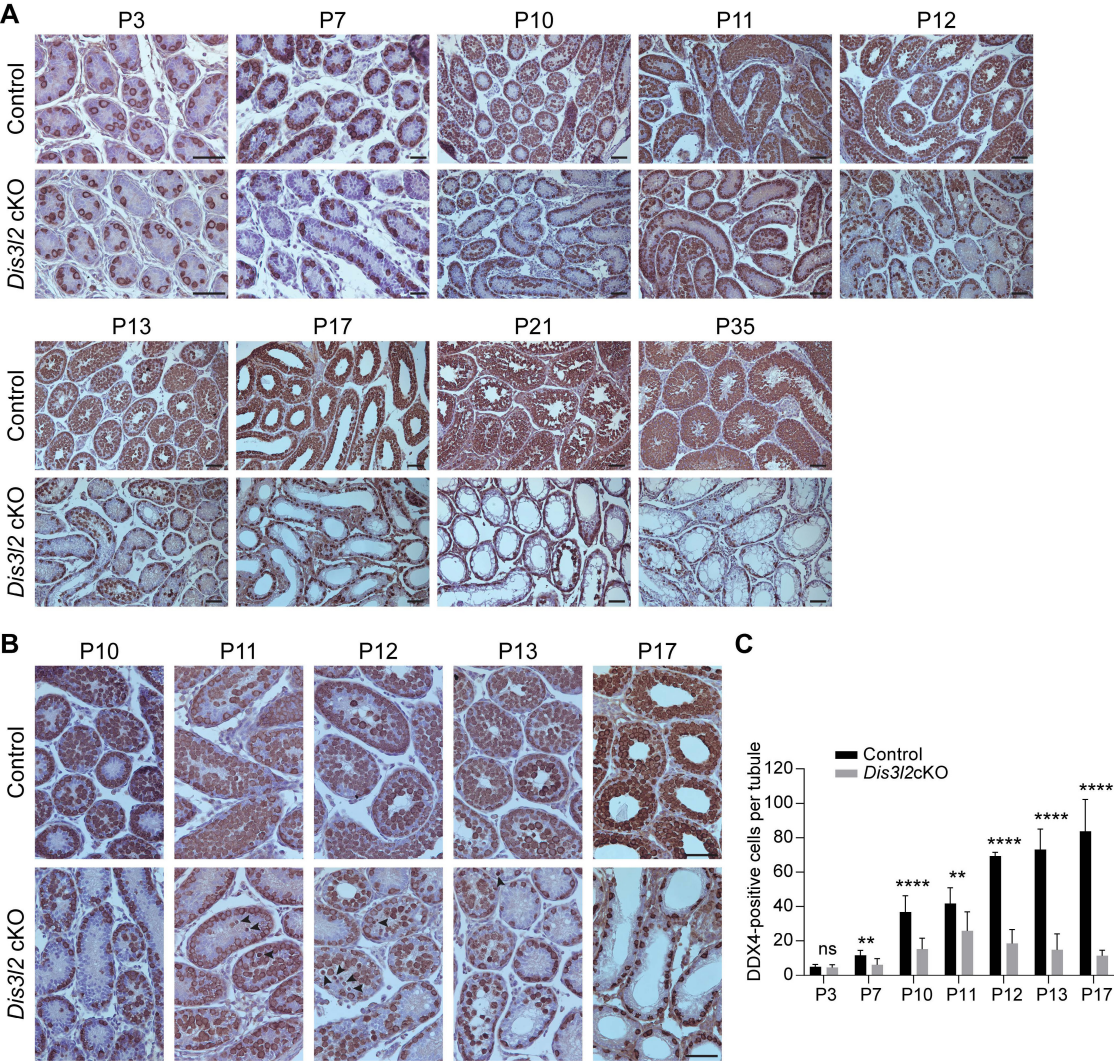
** DIS3L2 is essential for spermatocyte survival and the first wave of spermatogenesis.** (A) Histological and immunohistochemical analyses of DDX4 in the testicular tissue sections of control and *Dis3l2* cKO mice from P3 to P35. Scale bar, 50 μm. (B) Immunohistochemical analysis using a polyclonal DDX4 antibody was performed in the testis tissues of control and *Dis3l2* cKO mice from P10 to P17. Some of the images in (B) are enlarged from those in (A). Arrowheads indicate germ cells with pyknotic nuclei. Scale bar, 50 μm. (C) Statistical analysis of DDX4-positive cells per tubule in the testis tissue of control and *Dis3l2* cKO mice from P3 to P17. Mean ± s.d., n = 3 biologically independent testes from three different animals; ns, no significance, **P < 0.01, and ****P < 0.0001. Representative of n = 3 (A, B) independent biological replicates with similar results per condition.

To examine the earliest stage of spermatogenic defects, this study compared and analyzed testes isolated from P3 to P35 control and *Dis3l2* cKO mice using immunohistochemistry with an anti-DDX4 antibody. Immunohistochemical assessments revealed that *Dis3l2* cKO testes displayed normal germ cell numbers at P3 but exhibited a significant reduction in germ cell numbers by P7, indicating a defect in spermatogonial development (Figure 2A, C). From P10 to P35, control testes exhibited normal meiotic progression and completed the first wave of spermatogenesis (Figure 2A). Conversely, *Dis3l2* cKO testes showed reduced numbers of spermatocytes during the first wave of spermatogenesis (Figure 2A, C). In P21 and P35 *Dis3l2* cKO testes, DDX4-positive germ cells in the center of the seminiferous tubules were noticeably absent, leaving only a single layer of DDX4-positive germ cells at the epithelium (Figure 2A). This suggests defective spermatogenic lineage development, consistent with the defect observed in adult *Dis3l2* cKO testes. To delve deeper into the defects in spermatocyte development, testes from P10, P11, P12, P13, and P17 control and *Dis3l2* cKO mice were analyzed.

Histological and immunohistochemical results indicated that *Dis3l2* cKO spermatogonia were able to enter meiosis at P10, but experienced significant spermatocyte loss from P11 to P13 (Figure 2B). Pyknotic nuclei and shrunken cytoplasm of germ cells were frequently observed during this period (Figure 2B, arrowheads), indicating severe germ cell apoptosis. By as early as P17, germ cells in the central lumen of the seminiferous tubules had vanished in *Dis3l2* cKO testes (Figure 2B), reflecting impaired meiosis. Quantitative analysis of germ cell numbers further corroborated the drastic reduction of spermatogenic cells during meiosis (Figure 2C). In summary, these findings demonstrate the critical role of DIS3L2 in the development of primary spermatocytes and highlight that the ablation of DIS3L2 leads to substantial germ cell death and significant loss of spermatogenic cells during the first wave of spermatogenesis.

### DIS3L2 ablation impairs spermatogonial differentiation

**Figure 3:**
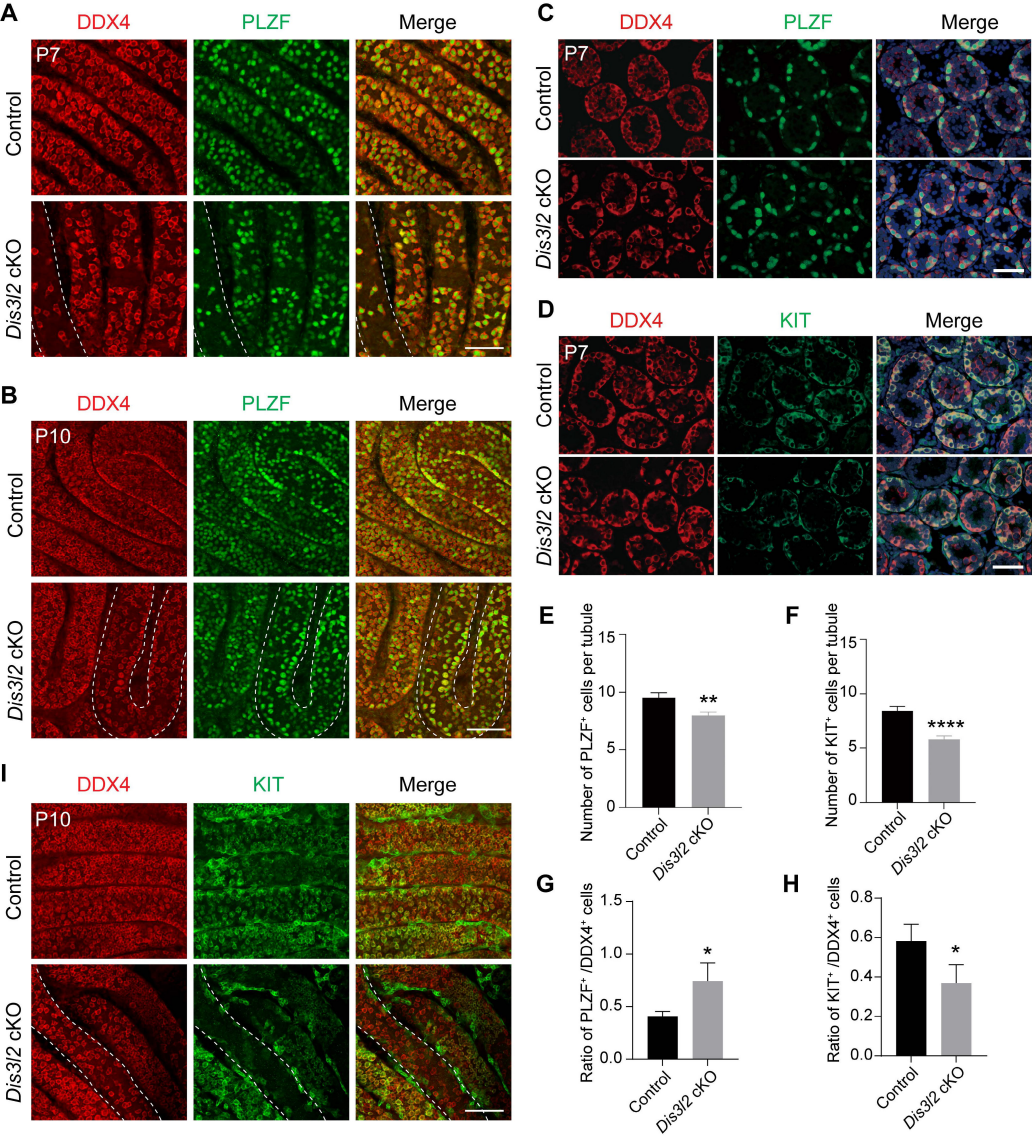
** DIS3L2 is required for spermatogonial development.** (A, B) Immunofluorescence of whole-mount testes from P7 (A) and P10 (B) control and *Dis3l2* cKO mice after staining with antibodies to DDX4 (left) or PLZF (middle) and merged (right). Scale bar, 50 μm. (C) Immunofluorescence staining of P7 control and *Dis3l2* cKO testicular sections with antibodies to DDX4 (left) or PLZF (middle) and merged with Hoechst 33342 to stain the DNA (right). Scale bar, 50 μm. (D) Same as (C), but with antibodies to DDX4 and KIT. (E, F) Statistical analysis of PLZF-positive cells (E) and KIT-positive cells (F) per tubule from P7 control and *Dis3l2* cKO testes. Mean ± s.d., n = 3 biologically independent testes from three different mice. **P < 0.01 and ****P < 0.0001. (G, H) Statistical analysis of the ratio of PLZF-positive cells to DDX4-positive cells (G) and KIT-positive cells to DDX4-positive cells (H) in P7 control and *Dis3l2* cKO testes. Mean ± s.d., n = 3 biologically independent replicates. *P < 0.05. (I) Whole-mount staining of testes from P10 control and *Dis3l2* cKO mice after staining with antibodies to DDX4 (left) or KIT (middle) and merged (right). Scale bar, 50 μm. Representative of n = 3 (A-D, I) independent biological replicates with similar results per condition.

*Stra8-Cre* induces the expression of the CRE enzyme in advanced germ cells before meiosis 23 and a significant decline of germ cells was observed in P7 *Dis3l2* cKO testes. Thus, spermatogonial development was examined. First, the overall number of germ cells and undifferentiated spermatogonia were determined by whole-mount staining using antibodies to DDX4 and PLZF. The number of DDX4-positive germ cells and PLZF-positive spermatogonia were significantly reduced in P7 and P10 *Dis3l2* cKO tubules (Figure 3A, B). This finding was confirmed by immunofluorescence staining in P7 testicular cross-sections that documented a significant reduction in the number of PLZF-positive cells per tubule in *Dis3l2* cKO testes compared with control testes (Figure 3C, E). Next, spermatogonial differentiation was investigated in *Dis3l2* cKO tubules. Immunofluorescence staining with antibodies targeting DDX4 and KIT documented that the number of KIT-positive spermatogonia per tubular cross-section was remarkably decreased in P7 *Dis3l2* cKO testes compared with controls (Figure 3D, F). Additionally, TUNEL-positive signals were significantly increased in *Dis3l2* cKO testes compared with the controls at P7 (Figure S4). Statistical analyses revealed a significant increase in the percentage of PLZF-positive cells and a dramatic decrease in the percentage of KIT-positive cells in P7 *Dis3l2* cKO tubules (Figure 3G, H), suggesting that DIS3L2 is required for spermatogonial differentiation. Whole-mount co-staining with antibodies to DDX4 and KIT demonstrated a significant decline in the number of KIT-positive cells in P10 *Dis3l2* cKO tubules (Figure 3I). Collectively, these findings indicate that conditional disruption of *Dis3l2* in pre-meiotic germ cells causes a remarkable decline in the number of spermatogonia, especially the differentiated spermatogonial population.

### DIS3L2 is required for spermatocyte survival and meiosis

**Figure 4:**
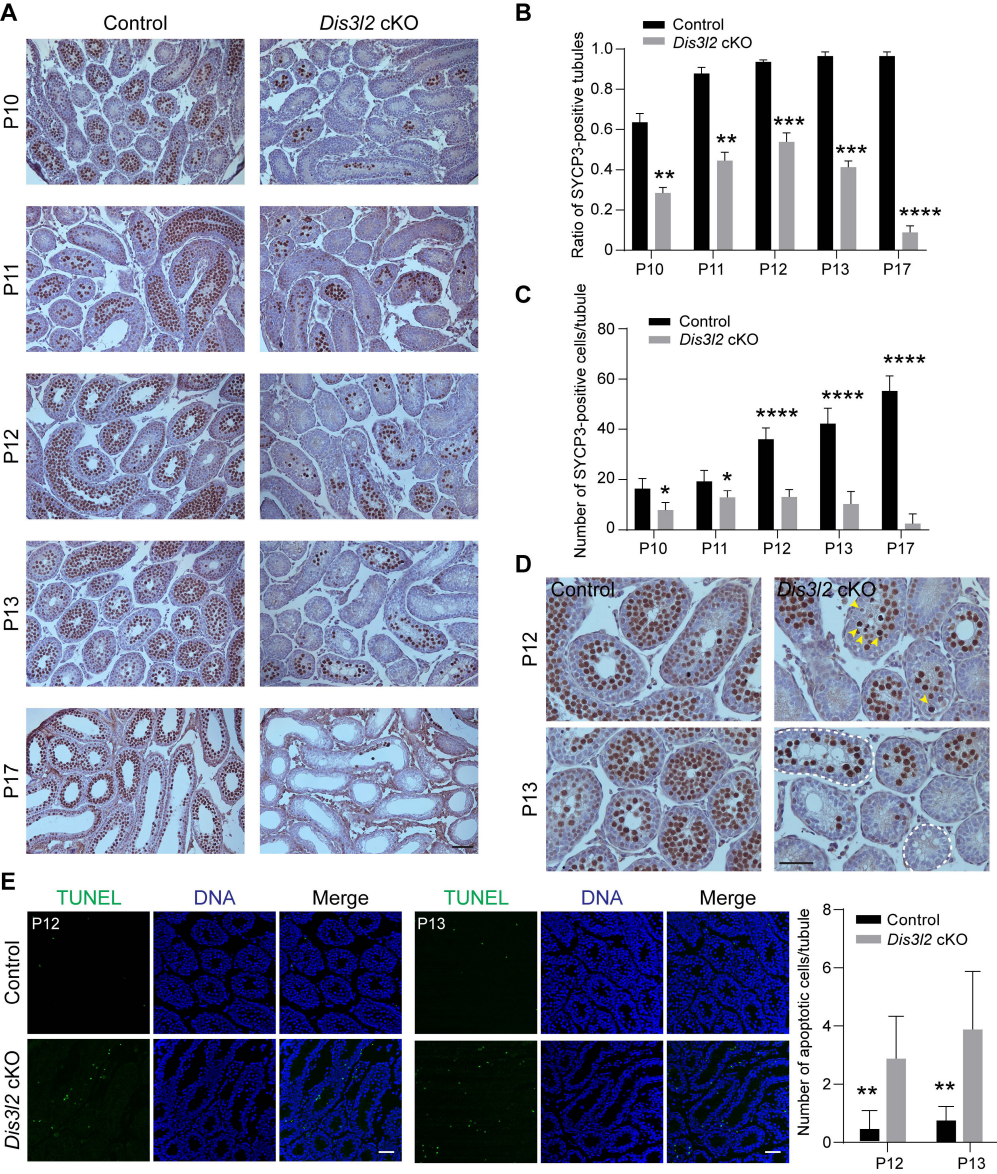
** DIS3L2 is required for male germ cell meiosis.** (A) Histological and immunohistochemical analyses of SYCP3 in the testis tissue of control and *Dis3l2* cKO mice from P10 to P17. Scale bar, 50 μm. (B) Statistical analysis of the ratio of SYCP3-positive tubules in the testis tissue of control and *Dis3l2* cKO mice from P10 to P17. Mean ± s.d., n = 3 biologically independent testes from three different mice. **P < 0.01, ***P < 0.001, and ****P < 0.0001. (C) Statistical analysis of the number of SYCP3-positive cells per tubule in the testis tissue of control and *Dis3l2* cKO mice from P10 to P17. Mean ± s.d., n = 3 biologically independent testes from three different mice. *P < 0.05, and ****P < 0.0001. (D) Immunohistochemical analysis of SYCP3 in the testis tissue of control and *Dis3l2* cKO mice at P12 and P13. Some of the images in (D) are enlarged from those in (A). Scale bar, 50 μm. Yellow arrowheads indicate spermatocytes with pyknotic nuclei. Dashed circles represent tubules with vacuoles. (E) TUNEL assay in the testis tissue of control and *Dis3l2* cKO mice at P12 and P13. DNA was stained with Hoechst 33342. Scale bar, 50 μm. The right panel shows the statistical analysis of the number of TUNEL-positive cells per tubule in the testis tissue of control and *Dis3l2* cKO mice at P12 and P13. Mean ± s.d., n = 3 biologically independent testes from three different mice. **P < 0.01. Representative of n = 3 (A, D, and E) independent biological replicates with similar results per condition.

To delve deeper into the meiotic defects in *Dis3l2* cKO testes, the expression pattern of the meiotic marker gene, synaptonemal complex protein 3 (SYCP3), a vital component of the synaptonemal complex, was examined from P10 to P17 using immunohistochemistry analysis. In control mouse testes, germ cells progressed through leptotene, zygotene, and pachytene, and eventually reached the diplotene stage during meiotic progression from P10 to P17 (Figure 4A). Conversely, SYCP3 exhibited a significantly reduced expression pattern in *Dis3l2* cKO testes (Figure 4A). Both the ratio of SYCP3-positive seminiferous tubules and the number of SYCP3-positive germ cells per tubule were markedly decreased in* Dis3l2* cKO testes compared with control testes from P10 to P17 (Figure 4B, C). Notably, an initial increase in the ratio of SYCP3-positive tubules and numbers of SYCP3-expressing cells per tubule from P10 to P12 was observed in *Dis3l2* cKO testes, followed by a substantial reduction from P13 to P17 (Figure 4B, C). By P17, very few SYCP3-positive cells were observed in the seminiferous tubules of *Dis3l2* cKO testes (Figure 4A). These results indicate that germ cells in *Dis3l2* cKO testes initiate meiosis and progress into the zygotene stage at P12 and P13, but subsequently undergo increased spermatocyte apoptosis and loss shortly afterwards. This was further corroborated by immunohistochemistry using SYCP3 and TUNEL assay (Figure 4D, E). Numerous small vacuoles were observed in the seminiferous tubules (marked by white dashed lines), and a few SYCP3-positive cells (highlighted by yellow arrowheads) displayed characteristic pyknotic nuclei in the centre of the seminiferous tubules in *Dis3l2* cKO testes at P12 and P13 (Figure 4D). TUNEL assays revealed a significant increase in apoptotic signals in *Dis3l2* cKO testes at P12 and P13 (Figure 4E). These findings unequivocally demonstrate that *Dis3l2* deficiency leads to severe spermatocyte loss, highlighting the essential role of DIS3L2 in normal meiosis.

### DIS3L2 is required for spermatocyte meiotic progression

**Figure 5:**
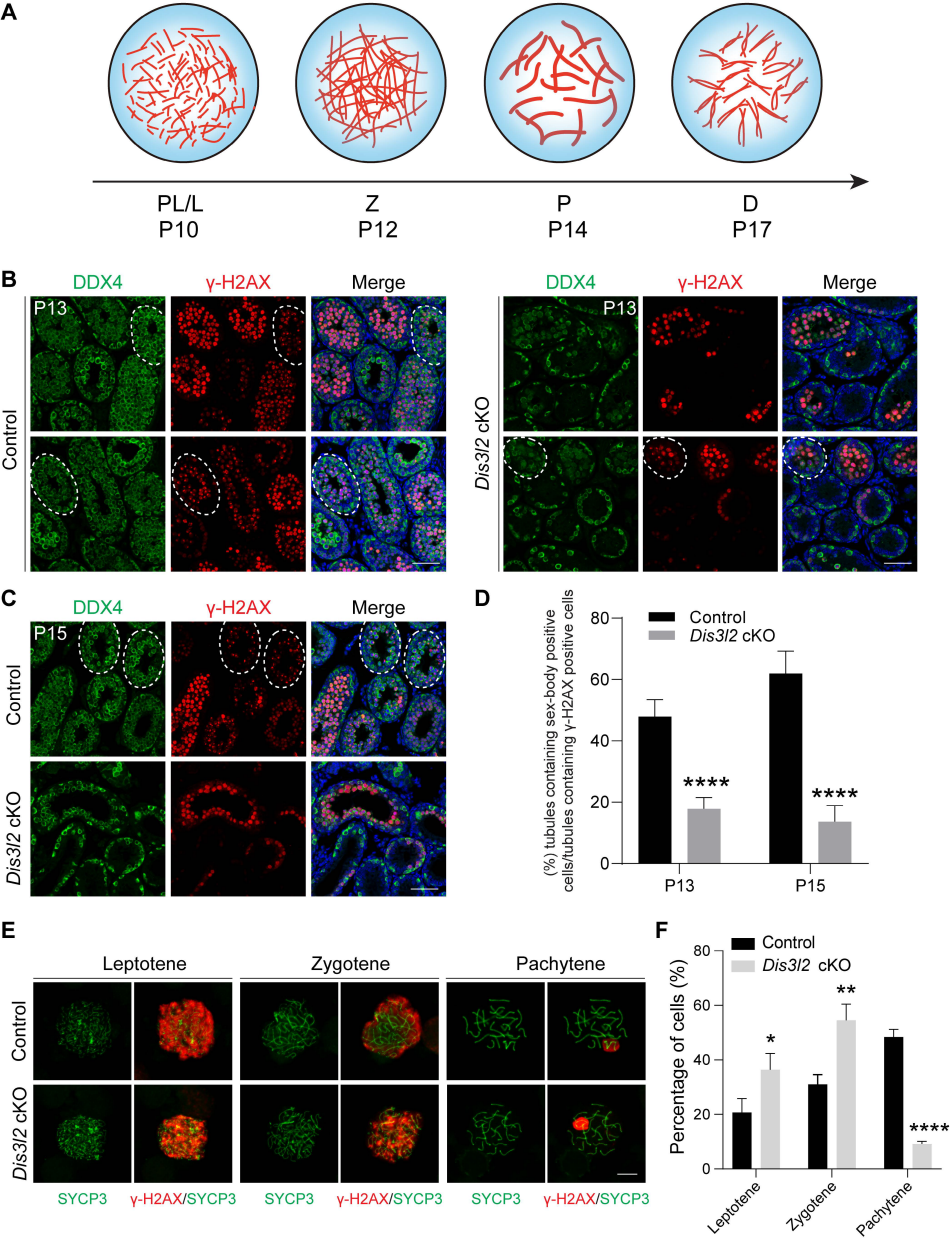
** DIS3L2 is required for spermatocyte meiotic progression.** (A) Schematic view of the four stages including pre-leptotene/leptotene (PL/L), zygotene (Z), pachytene (P), and diplotene (D) of meiotic prophase I corresponding to their developmental time point. (B) Immunofluorescence analysis results of P13 testes from control and *Dis3l2* cKO mice after co-staining with antibodies to DDX4 (germ cells) and γH2AX (spermatocytes) as well as Hoechst 33342 (DNA). Scale bar, 50 μm. Dashed circles indicate tubules containing sex-body-positive spermatocytes. (C) The same as (B), but at P15. (D) Statistical analysis of the ratio of tubules containing sex-body-positive spermatocytes in the testis tissue of control and *Dis3l2* cKO mice at P13 and P15. Mean ± s.d., n = 3 biologically independent testes from three different mice. ****P < 0.0001. (E) Chromosome spreads of spermatocytes from P13 control and *Dis3l2* cKO mice. Co-staining was performed with antibodies to SYCP3 and γH2AX. Scale bar, 10 μm. (F) Statistical analysis of the proportion of leptotene, zygotene, and pachytene spermatocytes in the P13 testis tissue of control and *Dis3l2* cKO mice. Mean ± s.d., n = 3 biologically independent testes from three different mice. *P < 0.05, **P < 0.01, and ****P < 0.0001. Representative of n = 3 (B, C, and E) biologically independent replicates with similar results per condition.

In male germ cells, meiosis is initiated around P10, with primary spermatocytes progressing through the stages of leptotene (P10), zygotene (P12), pachytene (P14), and reaching the diplotene stage at P17 (Figure 5A). γH2AX, a protein involved in double-strand break repair, exhibits robust expression in the nucleus from leptotene to zygotene stages and is enriched in the sex body (XY body) during the pachytene and diplotene stages. To characterize the meiotic deficiency in *Dis3l2* cKO testes, this study performed co-staining of DDX4 and γH2AX in seminiferous tubules at P13 and P15. Immunofluorescence staining revealed that control testes contained numerous sex-body positive cells and tubules at P13 and P15 (Figure 5B, C), indicating that a substantial number of spermatocytes had progressed into the pachytene stage and beyond. However, in *Dis3l2* cKO testes, sex-body positive spermatocytes were less prevalent in the tubules at P13 and nearly absent in the tubules at P15 (Figure 5B, C). The ratio of sex-body positive tubules was also significantly reduced in *Dis3l2* cKO testes compared with control testes at P13 and P15 (Figure 5D). The majority of spermatocytes in P13 and P15 *Dis3l2* cKO testes displayed strong γH2AX signals in the nuclei, indicating that leptotene/zygotene spermatocytes predominated in *Dis3l2* cKO testes and their transition to the pachytene stage and beyond was severely disrupted.

To precisely delineate the meiotic progression defects, this study prepared chromosome spreads of spermatocytes isolated from control and *Dis3l2* cKO mouse testes at P13 and stained them with SYCP3 and γH2AX antibodies.

Control testes harbored approximately 20.67%, 30.98%, and 48.35% of spermatocytes at the leptotene, zygotene, and pachytene stages, respectively (Figure 5E, F). While all three stages were observable in the chromosome spreads of *Dis3l2* cKO testes, only about 9.17% of spermatocytes were in the pachytene stage, with 36.33% and 54.50% of spermatocytes at the leptotene and zygotene stages, respectively (Figure 5E, F), suggesting impaired spermatocyte meiotic progression. In addition, chromosome synapsis was investigated and chromosome spreads of spermatocytes were prepared from P13 control and *Dis3l2* cKO testes. Co-staining of SYCP3 and synaptonemal complex protein 1 (SYCP1) documented that SYCP3 and SYCP1 signals were completely co-localized on the autosomes in control pachytene spermatocytes, while discontinued SYCP1 signals were frequently observed on the autosomes in *Dis3l2* cKO pachytene spermatocytes (Figure S5A), suggesting abnormal chromosome synapsis in *Dis3l2* cKO testes. Consistently, statistical analysis indicated that the proportion of abnormal synaptonemal pachytene spermatocytes was significantly increased in *Dis3l2* cKO testes compared with control testes (Figure S5B). Collectively, these results unequivocally demonstrate impaired transition of *Dis3l2*-deficient spermatocytes from the leptotene/zygotene stage to the pachytene stage and beyond, underscoring the essential role of DIS3L2 in normal spermatocyte meiotic progression.

### *Dis3l2* deficiency causes dysregulation of transcripts

**Figure 6:**
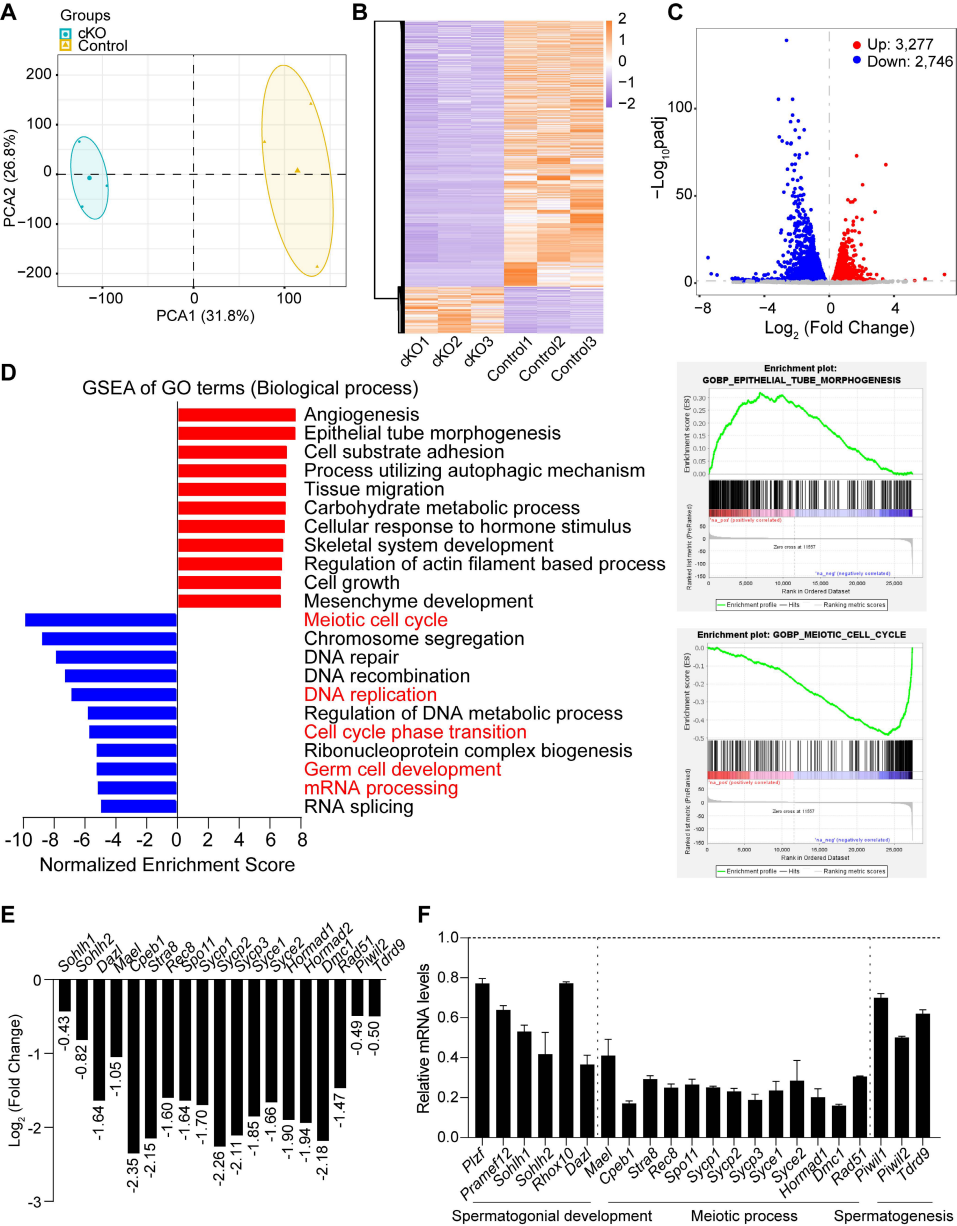
** DIS3L2 deletion disrupts the transcriptome in the testis tissue of *Dis3l2* cKO mice.** (A) Principal component analysis (PCA) of RNA-seq data from P12 control and *Dis3l2* cKO mice. (B) Heatmap of differentially expressed genes (DEGs) between control and *Dis3l2* cKO mice. (C) Volcano plot showing DEGs using an adjusted *P*-value of < 0.05 as the cutoff value by DESeq2. (D) Gene set enrichment analysis of biological process categories of *Dis3l2* cKO testes in comparison with control testes (left panel) using gene set enrichment analysis (GSEA) software program and molecular signatures database (MSigDB) C5 GO gene sets. Examples of the enrichment plot for Gene Ontology terms of epithelial tube morphogenesis and meiotic cell cycle (right panel). (E) RNA-seq results of selected transcripts (log_2_-fold change) related to the spermatogenic process in the testis tissue of *Dis3l2* cKO mice. (F) Quantitative RT-PCR assay for the validation of genes with downregulated expression involved in spermatogonial development, meiotic process, and spermatogenesis. For comparison, the abundance (relative to *β-actin*) of each gene in the testis tissue of control mice was set to 1. Data are presented as mean ± s.d. for n = 3 biologically independent samples per condition.

To investigate the molecular repercussions of *Dis3l2* depletion in spermatogenesis, bulk RNA-seq was performed in comparison with the transcriptome of control and *Dis3l2* cKO testes at P12. Sample clustering using principal component analysis (PCA) and the heatmap of differentially expressed genes (DEGs) revealed striking similarity among the three biological replicates within each condition, underlining the significance of the differences between the two conditions (Figure 6A, B), thereby affirming the reliability of the sequence data. The heatmap and volcano plot distinctly portrayed the transcriptional patterns between the two conditions and unveiled a series of DEGs (Figure 6B, C). The RNA-seq analysis pinpointed 6,023 DEGs, of which 3,277 transcripts were significantly upregulated and 2,746 genes were downregulated in *Dis3l2* cKO testes, with an adjusted *P*-value of < 0.05 as the cutoff, as determined by DESeq2 (Figure 6C). It was apparent that a large number of genes were dysregulated and the number of upregulated genes was higher than that of downregulated genes, suggesting defective RNA degradation in response to *Dis3l2* deletion. The DEGs were categorized into various biotypes to discern the potential targets of DIS3L2 in the testes. Notably, long intergenic noncoding RNA, processed transcript, antisense, processed pseudogene, and protein coding genes (mRNA) were the primary biotypes among the DEGs (Figure S6A). Collectively, these data indicate that conditional ablation of DIS3L2 ribonuclease causes significant dysregulation of transcripts in the testes.

Subsequently, gene set enrichment analysis (GSEA) was conducted to identify the enriched biological processes (gene ontology [GO] terms) and pathways in *Dis3l2* cKO testes. GO enrichment analysis revealed that upregulated transcripts were primarily associated with processes such as angiogenesis, epithelial tube morphogenesis, cell-substrate adhesion, tissue migration, carbohydrate metabolic process, skeletal system development, regulation of actin filament-based processes, cell growth, and mesenchyme development (Figure 6D). By contrast, downregulated transcripts were significantly enriched in GO terms related to biological processes such as meiotic cell cycle, chromosome segregation, DNA repair, DNA recombination, DNA replication, regulation of DNA metabolic process, ribonucleoprotein complex biogenesis, germ cell development, mRNA processing, and RNA splicing (Figure 6D). Consistently, pathway enrichment analysis revealed that processes such as lipid metabolism, Rho GTPase cycle, extracellular matrix organization, hemostasis, development biology, carbohydrate metabolism, cellular response to stimuli, and fatty acid metabolism were notably elevated. By contrast, processes such as cell cycle, DNA double-strand break repair, chromosome maintenance, DNA replication, RNA metabolism, mRNA processing, regulation of TP53 activity, and mRNA splicing were markedly reduced in *Dis3l2* cKO testes (Figure S6B).

The RNA-seq data highlighted a marked decrease in genes related to spermatogonial development, meiosis, and spermatogenesis in *Dis3l2* cKO testes (Figure 6E). This study further validated the expression patterns of these transcripts through real-time RT-PCR, confirming a significant downregulated expression of genes associated with spermatogonial development (e.g., *Plzf*, *Pramef12*, *Sohlh1*, *Sohlh2*, *Rhox10*, and *Dazl*), meiotic processes (e.g., *Mael*, *Cpeb1*, *Stra8*, *Rec8*, *Spo11*, *Sycp1*, *Sycp2*, *Sycp3*, *Syce1*, *Syce2*, *Hormad1*, *Dmc1*, and *Rad51*), and spermatogenesis (e.g., *Piwil1*, *Piwil2*, and *Tdrd9*; Figure 6F). This underscores that genes crucial for spermatogonial development, meiosis, and spermatogenesis are significantly downregulated in the absence of DIS3L2. Collectively, the RNA-seq data suggest that the loss of DIS3L2 leads to a severe impairment in RNA degradation, disrupting the delicate balance between RNA transcription and degradation. This disruption, in turn, leads to perturbations in RNA metabolism and the transcriptome during spermatogonial development and meiosis, ultimately culminating in impaired spermatogonial differentiation and spermatocyte meiotic progression.

### scRNA-seq defines the transcriptome of *Dis3l2* cKO spermatogonia

To determine the spermatogonial composition and transcriptome changes in *Dis3l2* cKO testes, single cells were isolated from P15 control and *Dis3l2* cKO testes, and scRNA-seq analysis was performed using the 10× Genomics platform. After quality control, a total number of 10,419 control and 8,731 *Dis3l2* cKO testicular cells were retained for subsequent analysis. Uniform Manifold Approximation and Projection (UMAP) and marker gene analyses were performed for cell type identification of the combined testicular cells. Eight cell types were identified based on the expression patterns of known marker genes in the mouse testis, including spermatogonia (SPG), spermatocytes (Scytes), and six somatic cell populations: endothelial cells, macrophages, myoid cells, Leydig cells, stromal cells, and Sertoli cells (Figure S7A, B). Statistical analysis revealed significant reductions in the cell number and percentage of both spermatogonia and spermatocytes in P15 *Dis3l2* cKO testes compared with control testes (Figure S7C, D), revealing abnormalities in spermatogonial development and spermatocyte meiosis in *Dis3l2* cKO testes.

**Figure 7:**
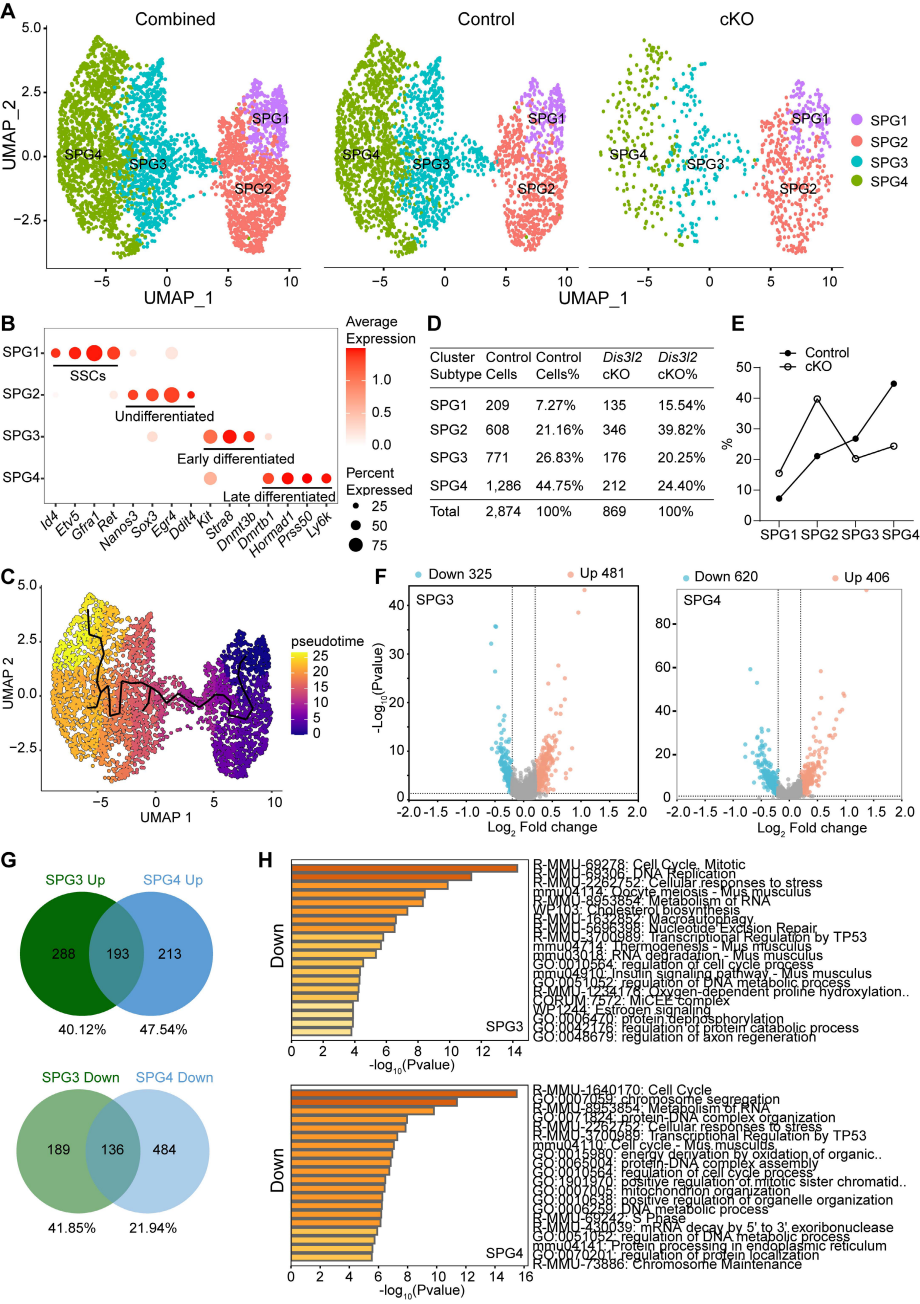
** scRNA-seq analysis of *Dis3l2* cKO spermatogonia.** (A) UMAP plots of combined (left panel), control (middle panel), and *Dis3l2* cKO (right panel) spermatogonia that had four distinct spermatogonial subtypes (SPG1, SPG2, SPG3, and SPG4). (B) Dot plot for the expression of selected marker genes across all identified cell types. (C) Pseudotime trajectory of the combined four spermatogonial subtypes. (D and E) Summary of detailed cell numbers and percentages of spermatogonia in each cell cluster in control and *Dis3l2* cKO testes. (F) Volcano plots of DEGs in *Dis3l2* cKO SPG3 (left panel) and SPG4 cells (right panel) using a cutoff of *P* < 0.05 and log_2_ fold-change of > 0.2. (G) Venn diagrams of upregulated genes (top panel) and downregulated genes (bottom panel) between SPG3 and SPG4 cells. (H) GO analysis of downregulated transcripts in the SPG3 subtype (top panel) and the SPG4 subtype (bottom panel) using Metascape.

To define how DIS3L2 ribonuclease affects cellular heterogeneity and transcriptome-wide signatures of spermatogonia, spermatogonia from P15 testes were re-clustered. After filtering out cells of poor quality, 3,743 spermatogonia were analyzed, and based on UMAP and marker gene analyses, four distinct spermatogonial subtypes (SPG1, SPG2, SPG3 and SPG4) were identified (Figure 7A, B). Based on the expression pattern of spermatogonial marker genes, SPG1 cells were assigned as SSCs, SPG2 as progenitor/undifferentiated spermatogonia, SPG3 as early differentiated spermatogonia, and SPG4 as late differentiated spermatogonia (Figure 7B). Monocle pseudotime analysis provided the developmental trajectory of spermatogonial cells from SPG1 to SPG4 (Figure 7C). The results revealed that 7.27%, 21.16%, 26.83%, and 44.75% cells were sorted into SPG1, SPG2, SPG3, and SPG4 subtypes, respectively, in control testes and 15.54%, 39.82%, 20.25%, and 24.40% cells were present in SPG1, SPG2, SPG3, and SPG4 subtypes, respectively, in *Dis3l2* cKO testes (Figure 7D, E). A remarkable increase in the percentage of the SPG1 and SPG2 subtypes and a decrease in the percentage of the SPG3 and SPG4 subtypes in *Dis3l2* cKO testes was observed. This indicates impairment of spermatogonial differentiation in the absence of DIS3L2 ribonuclease.

To uncover the underlying molecular causes of the perturbation in spermatogonial differentiation, differential gene expression analyses were conducted in SPG2, SPG3, and SPG4 cells. Using a cutoff of *P* < 0.05 and log_2_ fold-change > 0.2, a total of 519 DEGs, including 337 upregulated and 182 downregulated genes, were identified in *Dis3l2* cKO SPG2 cells, 806 DEGs (481 upregulated and 325 downregulated genes) in SPG3 cells, and 1,026 DEGs (406 upregulated and 620 downregulated) in SPG4 cells (Figure 7F and Figure S8A). A majority of genes were dysregulated in response to DIS3L2 ablation in SPG2, SPG3, and SPG4 cells, suggesting defective RNA degradation. GO analysis of DEGs in SPG2 cells revealed that upregulated genes were predominantly enriched in processes related to stem cell population maintenance, the respiratory electron transport chain, and cell division, while downregulated transcripts were primarily involved in DNA replication, cell cycle phase transitions, mRNA processing, and translation (Figure S8B). A large number of DEGs were overlapped between the SPG3 and SPG4 subtypes (Figure 7G), and the top 20 upregulated and downregulated transcripts of SPG3 and SPG4 subtypes were shown in Supplementary Figure S8C-E. In assessing the enriched GO terms in SPG3 cells, transcripts with increased abundance were primarily involved in oxidative phosphorylation, cell division, and mRNA metabolic processes (Figure S8C), and downregulated transcripts were significantly enriched in cell cycle, DNA replication, and metabolism of RNA (Figure 7H). Similar to SPG3, GO terms associated with energy-coupled proton transmembrane transport, cell cycle, mRNA metabolic processes, and translation were significantly enriched (Figure S8D), while terms related to the cell cycle and RNA metabolism were markedly decreased in the SPG4 subtype of *Dis3l2* cKO cells (Figure 7H). Collectively, these results imply that the downregulation of cell cycle and DNA replication genes may be directly related to the observed spermatogonial differentiation defect in *Dis3l2* cKO testes. Therefore, several genes related to spermatogonial differentiation and cell cycle in P8 control and *Dis3l2* cKO testes were examined. RT-qPCR analysis revealed that several spermatogonial differentiation genes (e.g., *Stra8*, *Sohlh1*, *Sohlh2*) and cell cycle genes (e.g., *Cenpa*, *Cdk2ap2*) were significantly decreased in *Dis3l2* cKO testes (Figure S9). Altogether, these findings suggest that alterations in spermatogonial differentiation genes and cell cycle genes upon loss of DIS3L2 ribonuclease may cause defective spermatogonial differentiation.

### Transcriptome signatures of *Dis3l2* cKO spermatocytes

**Figure 8:**
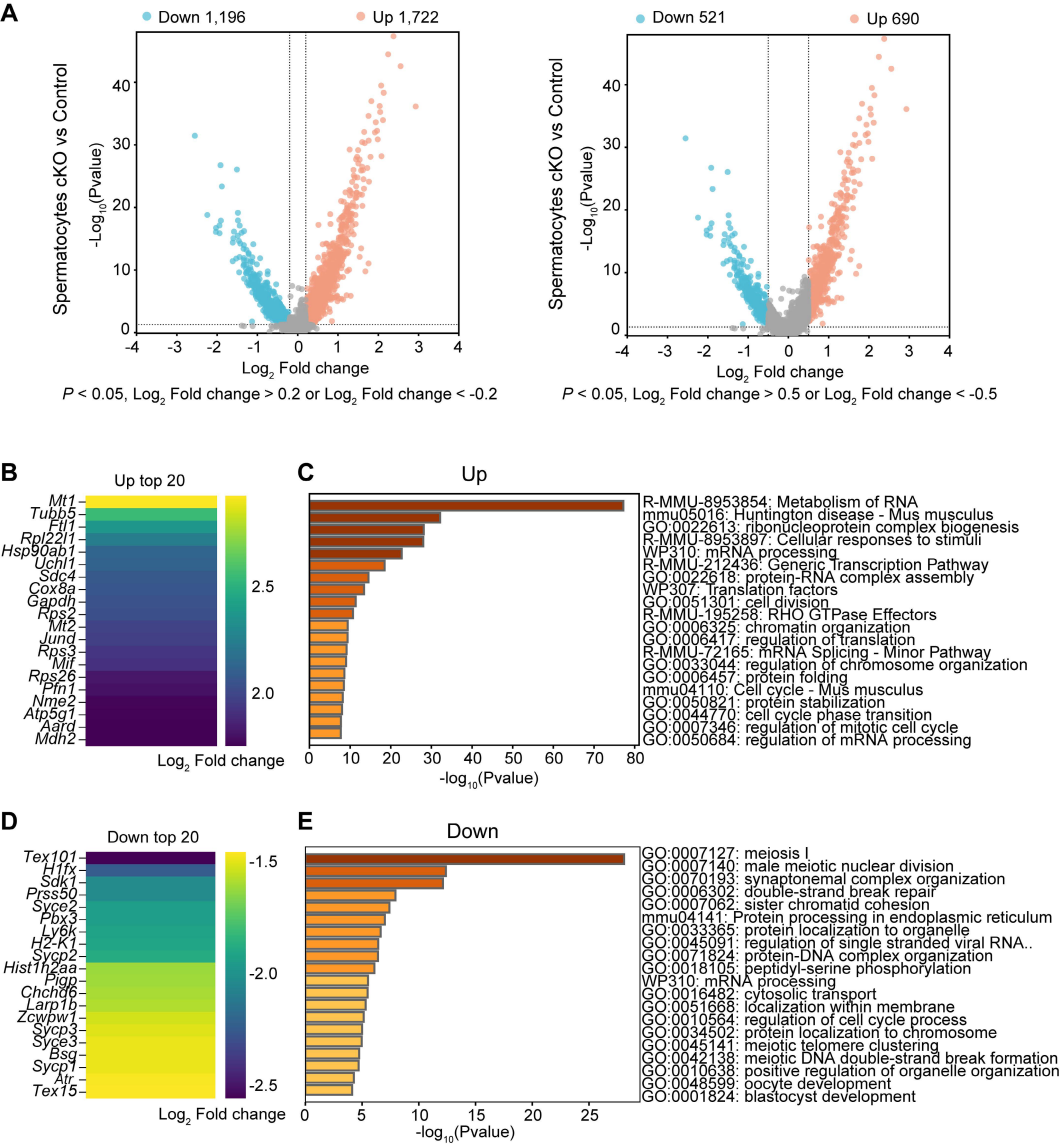
** Transcriptome signatures of *Dis3l2* cKO spermatocytes.** (A) Volcano plot of DEGs in *Dis3l2* cKO spermatocytes using a cutoff of *P* < 0.05 and log_2_ fold change of > 0.2 (left panel) and *P* < 0.05 and log_2_ fold change of > 0.5 (right panel). (B) Top 20 transcripts of upregulated DEGs. (C) Enriched GO terms and pathways of the upregulated genes analyzed using Metascape. (D) Top 20 downregulated transcripts. (E) Metascape analysis of GO terms and pathways for downregulated transcripts.

Differential gene expression analysis was performed on spermatocytes from scRNA-seq data and the enriched GO terms and pathways were determined in *Dis3l2* cKO spermatocytes. In total, 1,722 upregulated and 1,196 downregulated transcripts were identified in *Dis3l2* cKO spermatocytes using a cutoff of *P* < 0.05 and log_2_ fold-change of > 0.2. When restricted to a more stringent *P* < 0.05 and log_2_ fold-change of > 0.5, 690 genes were upregulated and 521 genes were downregulated (Figure 8A). The number of upregulated genes was higher than that of downregulated genes, suggesting insufficient RNA degradation upon DIS3L2 deletion. The top 20 of increased and decreased transcripts are shown in Figure 8B and D. GO analysis documented that increased transcripts were largely involved in the metabolism of RNA and regulation of translation, whereas downregulated genes were mainly enriched in meiosis I and mRNA processing in *Dis3l2* cKO spermatocytes (Figure 8C, E). The downregulation of meiotic genes (e.g., *Mael*, *Cpeb1*, *Stra8*, *Rec8*, *Spo11*, *Sycp1*, *Sycp2*, *Sycp3*, *Syce1*, *Syce2*, *Hormad1*, *Dmc1*, and *Rad51*) had been verified by RT-qPCR analysis in P12 *Dis3l2* cKO testes (Figure 6F). Immunoblot assay also confirmed the significant reduction of three meiosis-related proteins, namely RPA2, RAD51, and DMC1, in P12 *Dis3l2* cKO testes (Figure S10). Overall, these results indicate that *Dis3l2* deficiency in spermatocytes causes aberrant RNA metabolism, disrupted translation, and dysregulated meiotic genes, leading to impaired progression of spermatocyte meiosis.

### Comparison of RNA-seq data with scRNA-seq data

To further investigate the transcriptome changes resulting from *Dis3l2* depletion in male germ cells, bulk RNA-seq data and scRNA-seq data were compared and analyzed. As *Stra8-Cre*-mediated recombination starts from type A_1_ spermatogonia, we first compared the bulk RNA-seq data from testes with the scRNA-seq data from SPG3 cells. Among upregulated genes, 167 of 3,227 genes were shared among RNA-seq data (5.10%) and 481 genes among scRNA-seq data (34.72%) (Figure S11A). Among downregulated transcripts, 120 genes of a total of 2,746 genes were shared among RNA-seq data (4.37%) and 325 genes among scRNA-seq data (36.92%) (Figure S11A). GO analysis documented that the overlapped genes were significantly enriched for cell division, cell cycle, DNA replication, mRNA processing, and translation (Figure S11A). The abnormalities of these biological processes may directly relate to the observed defect in spermatogonial differentiation. Next, the bulk RNA-seq data from testes were compared with the scRNA-seq data from spermatocytes. The 531 increased transcripts that were shared represented 16.20% (3,277) and 30.84% (1,722), respectively, of the upregulated genes in the RNA-seq data and the scRNA-seq data (Figure S11B). Likewise, the 617 transcripts that were decreased in both datasets reflected 22.47% (2,746) and 51.59% (1,196) of downregulated genes in the RNA-seq data and the scRNA-seq data, respectively (Figure S11B). GO analysis revealed that the common DEGs were primarily enriched in meiosis I and male gamete generation (Figure S11B). The disruption of genes involved in meiosis and male gamete generation may be responsible for impaired meiotic progression of spermatocytes. Taken together, the disrupted genes involved in cell cycle, mRNA processing, translation, meiosis, and male gamete generation are possible indirect DIS3L2-targeted substrates and may account for the observed defects in spermatogonial differentiation and spermatocyte meiotic progression.

### Model of DIS3L2 in driving spermatogonial differentiation and meiotic progression

**Figure 9:**
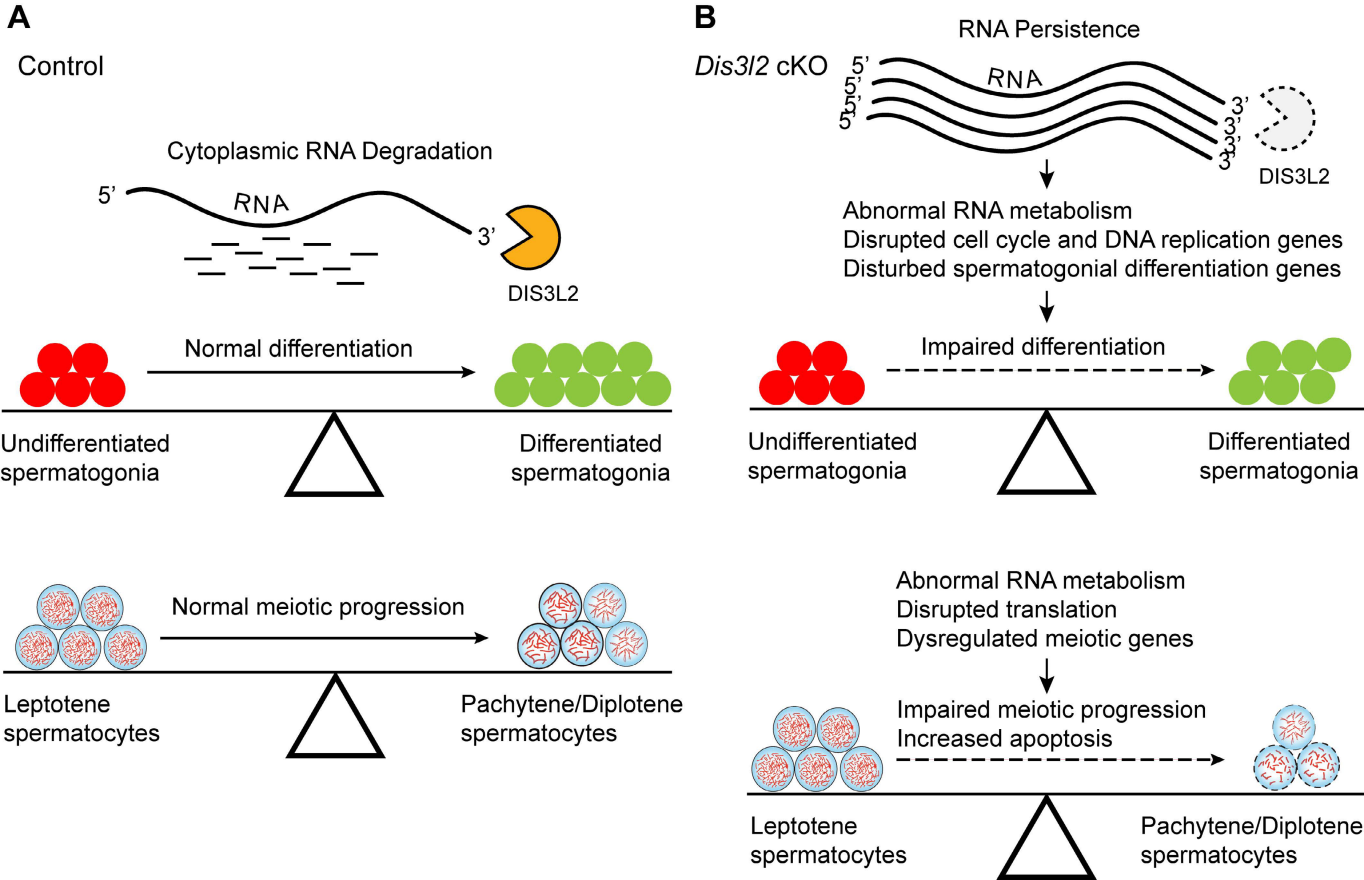
** Hypothetical model of DIS3L2 function in pre-meiotic and meiotic germ cells.** (A) DIS3L2-mediated RNA decay is required for normal spermatogonial differentiation and spermatocyte meiosis. (B) *Dis3l2* deficiency causes abnormal RNA metabolism, disrupted cell cycle genes, and disturbed spermatogonial differentiation genes, leading to impairment of spermatogonial differentiation. In spermatocytes, loss of DIS3L2 causes abnormal RNA metabolism, disrupted translation, and dysregulated meiotic genes, resulting in impaired meiotic progression and increased cell apoptosis.

The findings indicate that DIS3L2-mediated cytoplasmic RNA decay is essential for normal spermatogonial differentiation and spermatocyte meiotic progression (Figure 9A). Conditional disruption of *Dis3l2* in pre-meiotic germ cells causes defective RNA metabolism, downregulation of cell cycle genes, and aberrant expression of spermatogonial differentiation genes that impede spermatogonial differentiation (Figure 9B). In spermatocytes, *Dis3l2* deficiency results in abnormal RNA metabolism, aberrant translation, and disrupted meiotic genes that perturb spermatocyte meiotic progression and induce cell apoptosis (Figure 9B), leading to subsequent failure of spermatogenesis and male sterility.

## Discussion

Many investigations have been dedicated to elucidating the transcriptional networks essential for mouse spermatogenesis 4, 6, 25-27. Numerous RNases and co-factors have been identified as regulators of the correct transcriptome in mice, crucial for ensuring normal spermatogenesis 8-11. For instance, EXOSC10, the yeast homolog of RRP6, is a catalytic ribonuclease associated with the RNA exosome complex. It has been demonstrated to play a pivotal role in both male and female reproduction. In male mice, EXOSC10 is indispensable for germ cell development, and the conditional deletion of *Exosc10* in pre-meiotic germ cells results in spermatogenesis defects and subfertility 8. Disruption of RNA degradation in *Exosc10* conditional null oocytes leads to abnormalities in meiotic resumption and pre-implantation embryo development 28. Recently, DIS3L2 ribonuclease has been shown to degrade terminal-uridylated RNA, ensuring oocyte maturation and female fertility 22. However, whether DIS3L2 exerts a similar role in male spermatogenesis and fertility remains unclear.

The Perlman syndrome is often associated with a high neonatal mortality rate, and there are few reports of long-term survivors. Most of the infants with *DIS3L2* mutations develop respiratory distress and/or renal anomalies, and die within the few days of life 29. Only ten patients with *DIS3L2* mutations have been identified by the end of 2017, and most of them die before adulthood 29. Therefore, no relevant clinical findings regarding reproductive abnormalities with *DIS3L2* mutations have been reported to date. *Dis3l2^Δ11/Δ11^* mice (exon 11-deleted) display perinatal lethality, with no homozygous animals surviving the first postnatal day 30. Whether DIS3L2 is involved in germ cell development in males remains unknown. The present study generated a conditional *Dis3l2* KO mouse model using *Stra8-Cre* to investigate the function of DIS3L2 in spermatogenesis and male fertility. Our findings demonstrate that DIS3L2 ribonuclease is essential for normal spermatogenesis and male fertility. The conditional disruption of *Dis3l2* in pre-meiotic germ cells disrupts spermatogonial differentiation and impairs spermatocyte meiotic progression. Bulk RNA-seq data provide compelling evidence that perturbations in RNA degradation lead to disruptions in RNA abundance, thereby dysregulating gene expression associated with cell cycle in differentiated spermatogonia and meiosis in spermatocytes. scRNA-seq analysis further revealed that the absence of DIS3L2 in differentiated spermatogonia causes abnormal RNA metabolism and dysregulated cell cycle genes, and the loss of DIS3L2 in spermatocytes leads to aberrant RNA metabolism and disrupted meiotic genes. *Dis3l2* deficiency not only causes significant accumulation of transcripts but also results in the downregulation of numerous genes. Elevated RNA substrates or pathways in *Dis3l2* cKO may serve as repressors of gene expression. Therefore, the downregulated transcripts may be an indirect effect of the loss of DIS3L2 ribonuclease. Collectively, these disturbances ultimately result in defects in spermatogonial differentiation, meiotic progression, and subsequent spermatogenic lineage development.

Uridylation, the addition of uridine residues to the 3′ end of shortened poly(A) tails, has been associated with RNA degradation. DIS3L2 is known to selectively degrade uridylated RNAs, including uridylated pre-let-7 miRNAs and mRNAs 18, 31-33. TUT4 and TUT7 (collectively referred to as TUT4/7) are the primary cellular terminal uridylyltransferases responsible for mediating miRNA and mRNA 3′ uridylation 34-36. Previous reports have highlighted the crucial role of 3′ uridylation in shaping both male and female germline transcriptomes 37. TUT4/7-mediated 3′ uridylation is indispensable for timely maternal mRNA clearance and the maternal-to-zygotic transition 38. A recent study further underscores the significance of TUT4/7-mediated uridylation in clearing numerous transcripts expressed during the zygotene stage in pachytene spermatocytes. Deletion of both TUT4 and TUT7 in pre-meiotic germ cells by using *Stra8-Cre* mice leads to spermatogenic arrest in the late pachytene stage, accompanied by cell apoptosis, ultimately resulting in spermatogenic failure and male infertility 9. Gene expression analysis documents that a total number of 857 DEGs (732 upregulated and 125 downregulated genes) are identified in *Tut4/7* cKO pachytene spermatocytes, suggesting defective RNA decay when removing an RNA degradation signal by 3′ uridylation. TAIL-seq analysis further indicates that deficiency of TUT4/7 reduces terminal uridylation of genes not upregulated in the *Tut4/7* cKO pachytene spermatocytes by 5.8-fold, whereas the uridylation of upregulated transcripts is completely dependent on TUT4/7 9. This highlights the critical role of 3′ uridylation-primed mRNA degradation in facilitating male meiotic progression.

DIS3L2 exoribonuclease is responsible for targeting uridylated RNAs. The present study demonstrated that conditional ablation of *Dis3l2* in pre-meiotic germ cells similarly leads to defective meiotic progression, accompanied by cell apoptosis. The transition from leptotene/zygotene to the pachytene stage is significantly impeded by the loss of DIS3L2. Comparative bulk RNA-seq and scRNA-seq data provide evidence that the absence of DIS3L2 ribonuclease severely disrupts the transcriptome and the transcriptional expression pattern of genes related to meiosis. Differential gene expression analysis from scRNA-seq data identified 1,722 upregulated and 1,196 downregulated transcripts in *Dis3l2* cKO spermatocytes, suggesting defective RNA decay upon DIS3L2 deletion. These findings, in conjunction with prior research, affirm that TUT-DIS3L2-mediated RNA degradation is vital for meiotic progression during male germ cell meiosis. However, it is noteworthy that the deletion of DIS3L2 in spermatocytes results in an earlier defect in the transition from leptotene/zygotene to the pachytene stage, in contrast to *Tut4/7*-deficient spermatocytes that undergo arrest specifically at the pachytene stage. This implies that DIS3L2 may have functions independent of TUT4/7 in licensing meiotic progression.

The fate of uridylated transcripts is modulated by tail editing for either degradation by DIS3L2 or readenylation. A previous study reports that terminal oligo uridylation is often fewer than five residues in mouse oocytes through TAIL-seq analysis 37. Additionally, transcripts that have uridine at the 3′ termini are difficult to be isolated by the Poly(A) inclusive RNA isoform sequencing (PAIso-seq) method because the capture of RNA by PAIso-seq depends on the sequence of poly(A) tails. By employing various analyses such as Ribo-Minus RNA-seq, poly(A) RNA-seq, and PAIso-seq, a recent study indirectly investigates the fates of uridylated transcripts in *Dis3l2* cKO (*Zp3-Cre*; *Dis3l2^F/F^*) oocytes 22. Upon the depletion of DIS3L2 ribonuclease in mouse oocytes, uridylated-poly(A) RNAs accumulate significantly. Additionally, TUT4/7 uridylation may potentially elongate (from less than 5 to approximately 24 nucleotides) if not recognized and degraded by DIS3L2 ribonuclease. Furthermore, the uridylated transcripts in *Dis3l2* cKO oocytes can be re-adenylated using the same polyadenylation signal sequences as in control oocytes. Although the re-adenylated transcripts can recruit poly(A)-binding proteins, the length of the poly(A) tails of the uridylated-poly(A) RNAs is shorter, which may disrupt the recruitment of poly(A)-binding proteins and compromise the initiation of translation. Overall, uridylated-poly(A) RNAs exhibit increased stability and reduced translation in *Dis3l2* cKO oocytes. Our current study indicates that the transcriptome undergoes severe disruption upon DIS3L2 deletion. We speculate that uridylated-poly(A) RNAs accumulate and the global translation activity is compromised due to the features of the uridylated transcripts and the translation machinery proteins that have been insufficiently translated in *Dis3l2* cKO germ cells. However, whether the fates of uridylated transcripts in *Dis3l2*-deficient spermatogonia and spermatocytes show similar patterns to those in *Dis3l2* cKO oocytes needs to be further determined.

In *Dis3l2* cKO oocytes, several meiotic regulatory genes (e.g., *Rad21l*, *Meioc*, *Nr2c2*, *Sycp3*, and *Tubgcp6*) were dysregulated after DIS3L2 deletion 22. Some of these genes have known meiosis-related promoting or repressive roles 39-41. The disruption of meiosis genes and disrupted translation may cause impairment of meiotic resumption and germinal vesicle arrest in *Dis3l2* cKO oocytes. Similar dysregulations have been observed in *Dis3l2* cKO spermatocytes. The dysregulated transcripts were largely involved in RNA metabolism, translation regulation, and meiosis I in *Dis3l2* cKO spermatocytes. The results, together with previous findings, highlight the crucial role of DIS3L2 ribonuclease in safeguarding the transcriptome to ensure meiotic progression of mouse spermatocytes and oocytes necessary for fertility.

In summary, our investigations underscore that DIS3L2-mediated RNA degradation is indispensable for spermatogonial differentiation, spermatocyte meiotic progression, and male fertility. Upon DIS3L2 deletion, the transcriptome in differentiated spermatogonia and meiotic spermatocytes undergoes severe disruption, with notable dysregulation in cell cycle genes in spermatogonia and meiosis genes in spermatocytes. The data pinpoint DIS3L2 as a pivotal regulator in spermatogonial differentiation, male meiosis, and spermatogenesis, shaping the transcriptome during these critical developmental stages. Nevertheless, the precise molecular mechanisms governing the fates of uridylated transcripts in *Dis3l2*-deficient spermatogonia and spermatocytes warrant further investigation in future studies.

## Materials and methods

### Ethics statement

All animal procedures were conducted in accordance with the guidelines approved by the Ethics Committee for Animal Research of the School of Life Sciences, Shandong University, China (Approval No. SYDWLL-2021-90). The* Dis3l2* Floxed mice were procured from Cyagen, while the *Star8-Cre* mouse line 23 was generously provided by Professor Xiao-Yang Sun.

### Mouse genotyping

Mouse tail tissue samples were subjected to lysis with the Direct PCR Lysis Reagent containing proteinase K at 55 °C overnight. Subsequently, the samples were incubated at 85 °C for 1 hour to deactivate proteinase K. Specific DNA fragments were amplified using the EmeraldAmp GT PCR Master Mix (Takara Bio USA) along with appropriate primers. The PCR was conducted with an annealing temperature of 58 °C for 35 cycles using the Mastercycler Pro (Eppendorf). The primers employed for genotyping are detailed in Supplementary Table S1.

### Fertility assay

To evaluate fertility, one wild-type female mouse was housed together with either a control or a *Dis3l2* cKO male mouse for a period of 6 months. At least 3 mating cages were arranged for each genotype, and the average number of pups per litter was documented.

### Histology assay

Mouse testes and epididymides samples were immersed in Bouin's solution and left overnight at 4 °C. Subsequently, the samples were embedded in paraffin, sectioned (5 μm), and affixed onto slides before staining with periodic acid-Schiff (PAS) and hematoxylin. Finally, the samples were examined and captured under a microscope (Nexcope NE950).

### Immunohistochemistry and immunofluorescence

Following de-waxing, rehydration, and antigen retrieval using 0.01% sodium citrate buffer (pH 6.0), sections were blocked with blocking buffer containing 0.05% Tween-20 at room temperature for 1 hour, followed by an overnight incubation with primary antibodies (Supplementary Table S2) at 4 °C. For immunohistochemistry (IHC), the ImmPRESS™ Polymer Detection Kit (Vector Laboratories) and diaminobenzidine (DAB) were utilized to visualize antibody binding. For immunofluorescence (IF) staining, specific secondary antibodies (Supplementary Table S2) were employed to detect the antigen, and DNA was counterstained with Hoechst 33342. Images were captured with a fluorescent microscope (Nexcope NE950).

For whole-mount staining, freshly isolated tissues were fixed in 4% paraformaldehyde (PFA) overnight at 4 °C, permeabilized in blocking buffer containing 5% donkey serum, 3% bovine serum albumin, 0.5% Triton X-100, and 0.05% Tween 20 overnight at 4 °C and then incubated with primary antibodies (Supplementary Table S2) overnight at 4 °C. Secondary antibodies (Supplementary Table S2) conjugated to Alexa Fluor were incubated overnight at 4 °C. After washing in phosphate-buffered saline (PBS), samples were mounted with PBS on the slides. Fluorescence images were captured by confocal microscopy (Leica STELLARIS 5).

### *In situ* hybridization

The sequence of the mouse *Dis3l2* probe for *in situ* hybridizations was retrieved from the GenePaint database. Templates were generated via PCR from cDNA and subsequently subcloned into the pEASY Blunt Zero Cloning vector. This construct was then linearized and utilized as a template for in vitro transcription using T7 polymerase to produce a probe labeled with digoxigenin-11-UTP (Roche). Testis samples freshly isolated were fixed in 4% PFA overnight at 4°C, followed by a wash in PBS. Subsequently, the samples underwent sequential incubation in 15% and 30% sucrose in PBS at 4°C for 16 hours and were then embedded in Tissue-Tek O.C.T. on dry ice. Sections, 10 μm thick, were cut and air-dried at 37°C for 30 minutes. After treatment with 2 μg/ml proteinase K, the slices were incubated at 65°C overnight with the digoxigenin-labeled probe in a hybridization solution containing 50% (v/v) formamide, 5 × saline sodium citrate, 5 × Denharts, 250 μg/ml yeast RNA, and 500 ug/ml herring sperm DNA. The slides were subsequently washed in 0.2 × saline sodium citrate at 62°C, followed by an incubation with anti-digoxigenin antibody (Roche) at 4°C overnight. Signals were developed in NBT/BCIP stock solution (Roche) and captured using a fluorescent microscope (Nexcope NE950).

### Meiotic chromosome spreads

Mouse testes were decapsulated, placed in hypotonic buffer (30 mM Tris pH 7.5, 17 mM trisodium citrate, 5 mM EDTA, and 50 mM sucrose) for 30 minutes, and seminiferous tubules were chopped to release germ cells in 200 mM sucrose. The resulting cell suspensions were deposited on slides pre-coated with fixation buffer (1% PFA and 0.1% Triton-X100 in PBS), fixed for 3 hours in a humidifying chamber at room temperature, and air-dried. For immunostaining, the slides were incubated with primary antibodies (Supplementary Table S2) at 4°C overnight and subsequently with Alexa Fluor secondary antibodies (Supplementary Table S2) for 1 hour at room temperature. Images were captured using a fluorescent microscope (Nexcope NE950).

### Immunoblot assay

Total protein was extracted in 1 × LDS sample buffer with 1× NuPAGE Sample Reducing Agent (Thermo Fisher Scientific). Proteins were separated on 4%-12% Bis-Tris gels and electrophoretically transferred onto polyvinylidene difluoride membranes. The membranes were blocked with 5% nonfat milk in Tris-buffered saline containing 0.05% Tween 20 (TBST) at room temperature (RT) for 1 h and probed with primary antibodies (Supplementary Table S2) overnight at 4 °C. The membranes were washed three times with TBST and incubated (1 h, RT) with secondary antibodies (Supplementary Table S2), followed by washing with TBST and developed using SuperSignal West Dura Extended Duration Substrate (Thermo Fisher Scientific). Signals were detected with Hyperfilm ECL (GE Healthcare) according to the manufacturer's instructions.

### RNA isolation and RT-qPCR

Total RNA was extracted from mouse testes using the AFTSpin Tissue/Cell Fast RNA Extraction Kit for Animals (ABclonal), and cDNA was synthesized with the SuperScript IV First-Strand Synthesis System (Thermo Fisher Scientific). The RT-qPCR assay was conducted using the iTaq Universal SYBR Green Supermix (Bio-Rad) on the QuantStudio 6 Flex Real-Time PCR System (Thermo Fisher Scientific). The primers utilized in these experiments are detailed in Supplementary Table S3. The relative abundance of each transcript was calculated using 2^-ΔΔCt^ and normalized to endogenous* β-actin* expression 42.

### RNA-seq library preparation

Total RNA was extracted using the RNeasy Micro Kit, and libraries were prepared using a Universal RNA-seq Library Preparation Kit (TECAN) according to the manufacturer's instructions. Briefly, double-strand cDNA was synthesized using a mixture of random and poly(T) primers, followed by double-stranded cDNA fragmentation, end repair, adaptor ligation, strand selection, targeted transcript depletion with AnyDeplete, and PCR amplification. The final PCR-amplified libraries were sequenced on an Illumina HiSeq 2500 platform, generating 50-bp single-end reads.

### RNA-seq analysis

The processed reads were aligned to the University of California Santa Cruz (UCSC) mm10 reference genome through the use of HISAT2. Subsequently, the quantification results obtained from "featureCount" were subjected to analysis with the Bioconductor package DESeq2, which applies a negative binomial distribution to estimate both technical and biological variability. A PCA plot was generated to identify any potential sample outliers. Comparative analyses were performed between cKO and control samples. A gene was considered differentially expressed when the false discovery rate (FDR) adjusted *p*-value for differential expression was less than 0.05. The correlation heatmap was created using the R software package with the ''ggplot2'' package. GSEA was performed using GSEA v4.3 software program. Genes were ranked before analysis based on their fold change in gene expression. This application assigns scores to a sorted list of genes regarding their enrichment in selected functional categories (Kyoto Encyclopedia of Genes and Genomes and GO). The significance of the enrichment score was evaluated through 1000 permutations. Benjamini and Hochberg's FDR was computed to account for multiple testing adjustments. A *q*-value of < 0.05 was considered statistically significant.

### scRNA-seq library preparation

scRNA-seq libraries were prepared using Chromium Next GEM Single Cell 3′ Reagent Kits v3.1 (10× Genomics) according to the manufacturer's instructions. Briefly, single cells obtained from digested testes were mixed with a suspension containing barcoded beads and unique molecular identifier (UMI) elements that allow specific tagging of messenger RNA. After partitioning thousands of cells into nanoliter-scale Gel Bead-In-EMulsions (GEMs) and barcoding, full-length barcoded cDNA was then amplified by PCR to generate sufficient mass for library construction. Libraries were constructed by fragmentation, end repair, A-tailing, adaptor ligation, and index PCR. After ensuring adequate quality of cDNA libraries, the samples were sequenced on an Illumina NovaSeq 6000 platform at Guangzhou Gene Denovo Biotechnology Co., Ltd.

### scRNA-seq data processing

Raw sequencing reads were carried out using the Cell Ranger Single-Cell Software Suite (10× Genomics) for tasks such as alignment, filtering, UMI quantification, and generation of the gene-barcode matrix. The raw gene expression matrices produced per sample using CellRanger were imported into R (version 4.3.0) and converted to a Seurat object utilizing the Seurat R package (version 4.3.0.1) 43. The filtration process eliminated deceased cells, doublets, and low-quality cells by considering total UMIs, total genes, and the percentage of mitochondrial UMIs. Subsequently, quality filters were set to retain cells that had 1) ≥ 200 and ≤ 8000 uniquely expressed genes; 2) ≥ 1000 UMIs; 3) ≤ 20% of reads mapped to the mitochondrial genome; and 4) ≤ 5% of reads mapped to hemoglobin genes, as these cells likely depict contaminating erythrocytes.

### Data integration and major cell type determination

Cells from control and *Dis3l2* cKO samples were integrated, and batch effects were corrected using Harmony from Seurat 44. Normalization was applied to each sample, and cell cycle scoring was conducted using Seurat. Highly variable genes (HVGs) were identified using *SCTransform*, with mitochondrial genes being excluded. Subsequently, scaled PCA was performed on the filtered HVGs, and UMAP was used for dimensionality reduction. A shared nearest neighbor graph was constructed, followed by clustering at a resolution of 1.6 in Seurat. The canonical marker genes were applied to annotated cell clusters to identify biological cell types.

### Identification of marker genes and differentially expressed genes

Marker genes for cell types were identified by comparing expression levels in the cluster of interest against other clusters using the Seurat *FindMarkers* function (MAST, version 1.26.0) 45. Marker genes were defined based on the following criteria: (1) the average expression value in the cluster of interest was at least 2.5-fold higher than that in the rest of the clusters; (2) marker genes should be detectable in at least 10% of the cells in the cluster of interest; and (3) marker genes should have the highest mean expression in the cluster of interest compared with the rest of the clusters. Differentially expressed genes between the two samples were computed using the Seurat *FindMarkers* function with parameters set as min.pct = 0.01 and logfc.threshold = 0.01. GO analysis was conducted using Metascape 46 to determine the biological processes and pathways.

### Pseudotime analysis

Pseudotime analysis was performed using monocle 3 (Version 1.0.0) 47. Seurat object for cell types was converted to Monocle object and metadata and UMAP embedding were passed to Monocle object as well. Data were processed using the “preprocess_cds” function. Cells were clustered using the “cluster_cells” function with cluster_method = “leiden”. The trajectory graph was learned with the “learn_graph” function and cells were ordered in pseudotime with “order_cells” function.

### Statistical analysis

Data are presented as the mean ± s.d. Statistical analysis was conducted using GraphPad Prism. The variances of the two groups were compared using the two-tailed Student's t-test, and significance was defined as ns (no significance), *P < 0.05, **P < 0.01, ***P < 0.001, and ****P < 0.0001.

## Supplementary Material

Supplementary figures and tables.

## Abbreviations

cKO: conditional knockout
RT-PCR: Reverse transcription-polymerase chain reaction
RT-qPCR: real-time quantitative PCR
PAS: periodic acid-Schiff
DDX4: DEAD-box helicase 4
WT1: Wilms tumor 1
PLZF: promyelocytic leukemia zinc finger
SYCP3: synaptonemal complex protein 3
SYCP1: synaptonemal complex protein 1
TUNEL: terminal-deoxynucleotidyl transferase mediated nick end labelling
DEGs: differentially expressed genes
GSEA: gene set enrichment analysis
GO: gene ontology
UMAP: uniform manifold approximation and projection
PCA: principal component analysis
SSCs: spermatogonial stem cells
SPG: spermatogonia
Scytes: spermatocytes
TUT4: terminal uridylyl transferase 4
TUT7: terminal uridylyl transferase 7
PAIso-seq: Poly(A) inclusive RNA isoform sequencing
scRNA-seq: single cell RNA sequencing
IF: immunofluorescence
IHC: immunohistochemistry
GFRA1: GDNF family receptor alpha 1
KIT: stem cell growth factor receptor
DIS3L2: DIS3-like exonuclease 2
