# Supplementary material for: Conditional ablation of DIS3L2 ribonuclease in pre-meiotic germ cells causes defective spermatogenesis and infertility in male mice
Source: Theranostics. 2024 Sep 3;14(14):5621–42. doi: 10.7150/thno.98620 (PMC11413780; doi:10.7150/thno.98620)
Supplement: Supplementary file 1 — Supplementary figures and tables. [file thnov14p5621s1.pdf]

Supplementary material

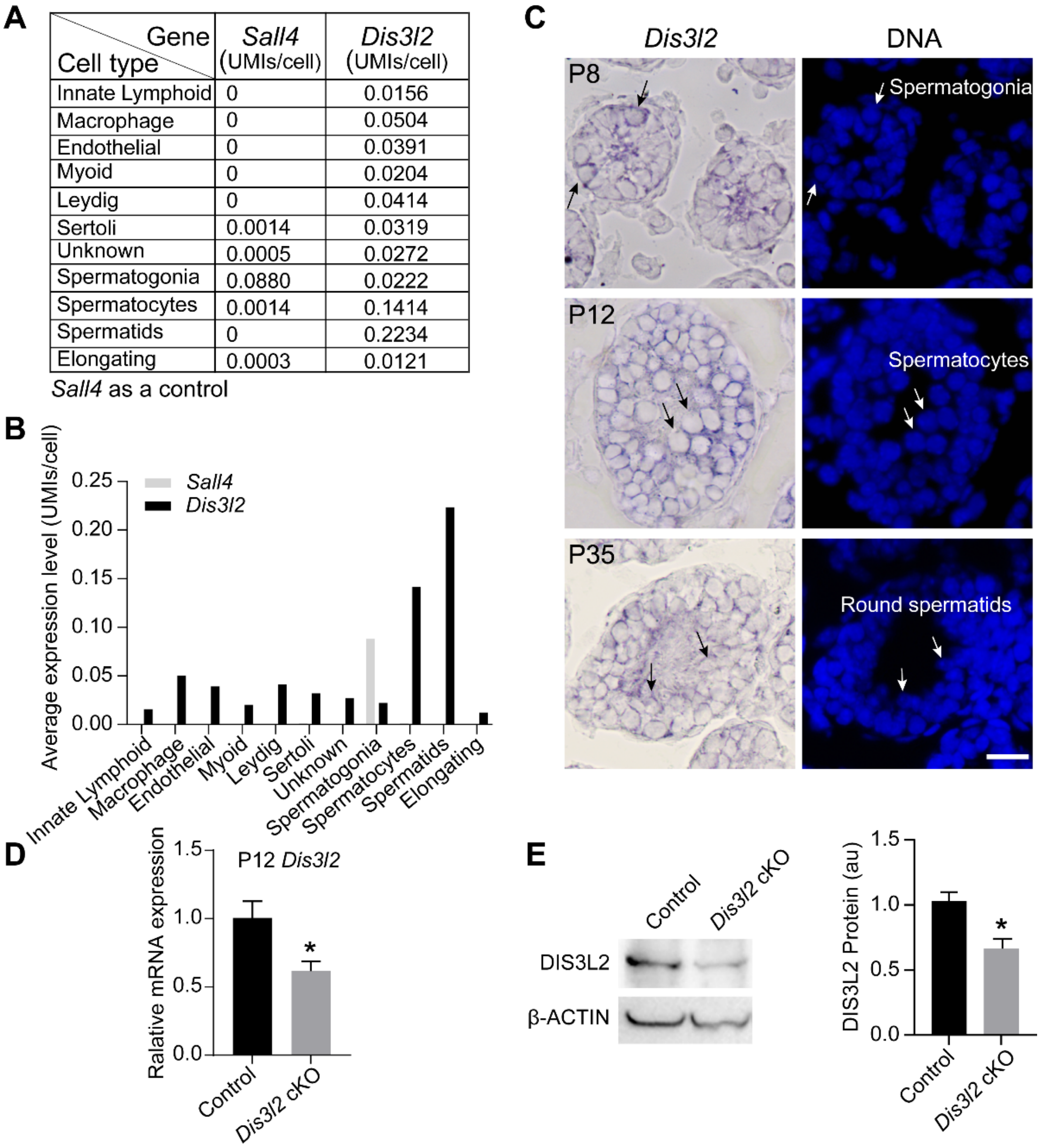

Figure S1. *Dis3l2* transcript expression in the mouse testes. (A and B) Analyses of the abundance of *Dis3l2* transcripts in the 11 testicular cell types based on previously reported scRNA-seq data in adult mouse testes. Numbers in A and the Y axis in B reflect the averaged UMIs detected in individual cells. (C) *In situ* hybridization mRNA analysis of *Dis3l2* in P8, P12, and P35 wild-type mouse testes. The DNA was stained with Hoechst 33342. Scale bar, 20 μm. Representative

of n = 3 biologically independent replicates with similar results per condition. (D) Quantitative RT-PCR analysis of *Dis3l2* in P12 control and *Dis3l2* cKO testes. For comparison, the abundance (relative to *β-actin*) of *Dis3l2* in control testes was set to 1. Data are presented as mean ± s.d for n=3 biologically independent samples per condition. *$P < 0.05$. (E) Immunoblot assay of DIS3L2 protein in P12 control and *Dis3l2* cKO testes using β-ACTIN as a load control (left panel) and the relative DIS3L2 protein expression in P12 control and *Dis3l2* cKO testes (right panel). Mean ± s.d, n=3 biologically independent replicates with similar results per condition. *$P < 0.05$.

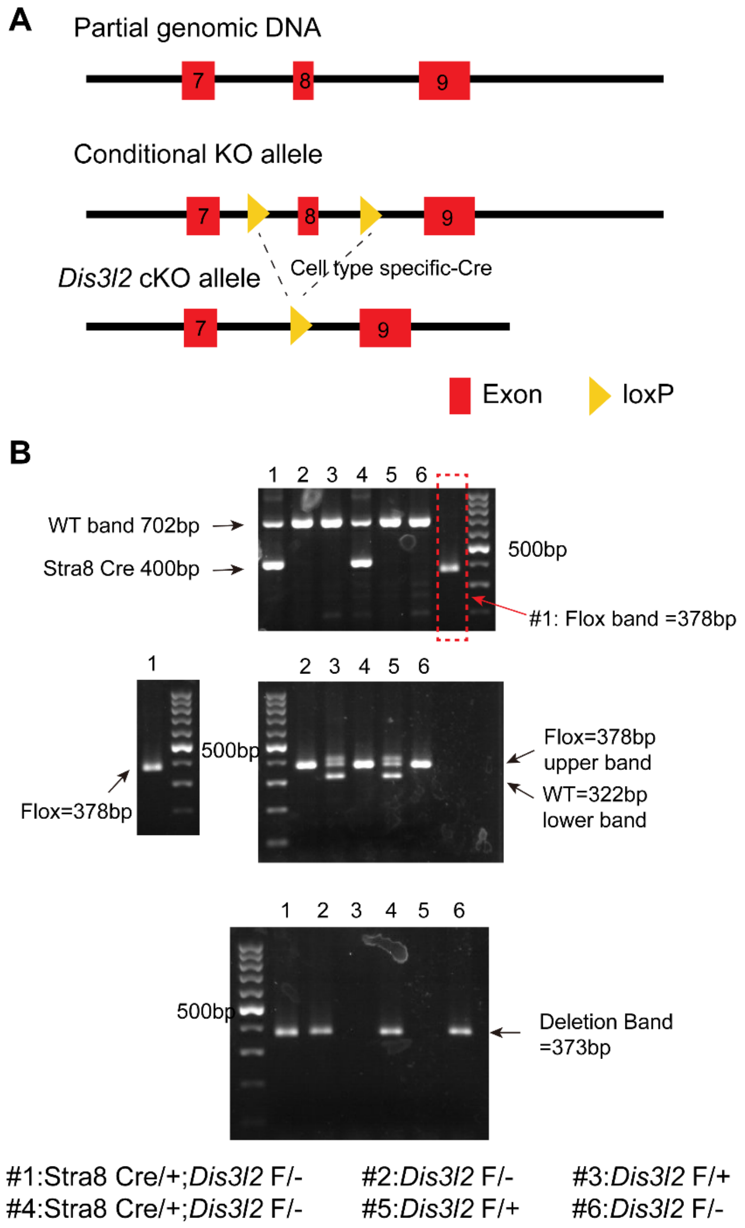

Figure S2. Establishment of *Dis3l2* cKO mice. (A) Exon map of the mouse *Dis3l2* locus. Two loxP sites were designed flanking exon 8. Exon 8 was deleted after crossing with the *Stra8-Cre* line, leading to a frameshift mutation of the *Dis3l2* gene. (B) An example of the polymerase chain reaction genotyping results to establish the *Dis3l2* cKO mice. The molecular mass and the sizes of the DNA bands are indicated.

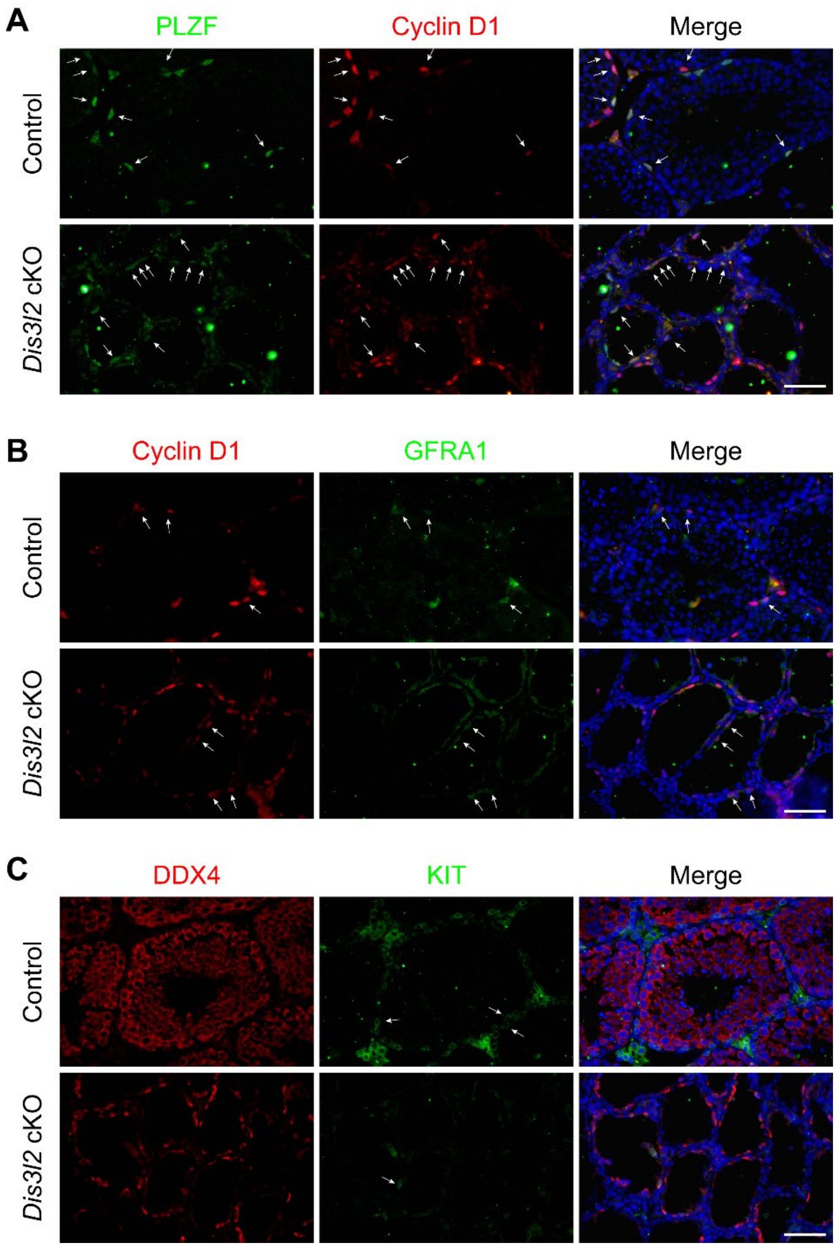

Figure S3. Immunohistochemical analysis of adult *Dis3l2* cKO testes. (A) Immunofluorescence staining of adult control and *Dis3l2* cKO testes with antibodies to PLZF (left) or Cyclin D1 (middle), and merged with Hoechst 33342 to stain DNA (right). Arrows indicate both PLZF- and Cyclin D1-positive cells. Scale bars: 50 μm. (B) Co-immunostaining of Cyclin D1 (left), or GFRA1 (middle), and merged with Hoechst 33342 to stain DNA (right) in adult control and *Dis3l2* cKO testes. Arrows indicate both Cyclin D1- and GFRA1-positive cells. Scale

bars: 50 µm. (C) Immunofluorescence of cross-sections from adult control and *Dis3l2* cKO testes after staining with antibodies to DDX4 (left), or KIT (middle), and merged with Hoechst 33342 to stain DNA (right). Arrows indicate KIT-positive cells. Scale bars: 50 µm. (A-C) Images are representative of three independent biological replicates with similar results per condition.

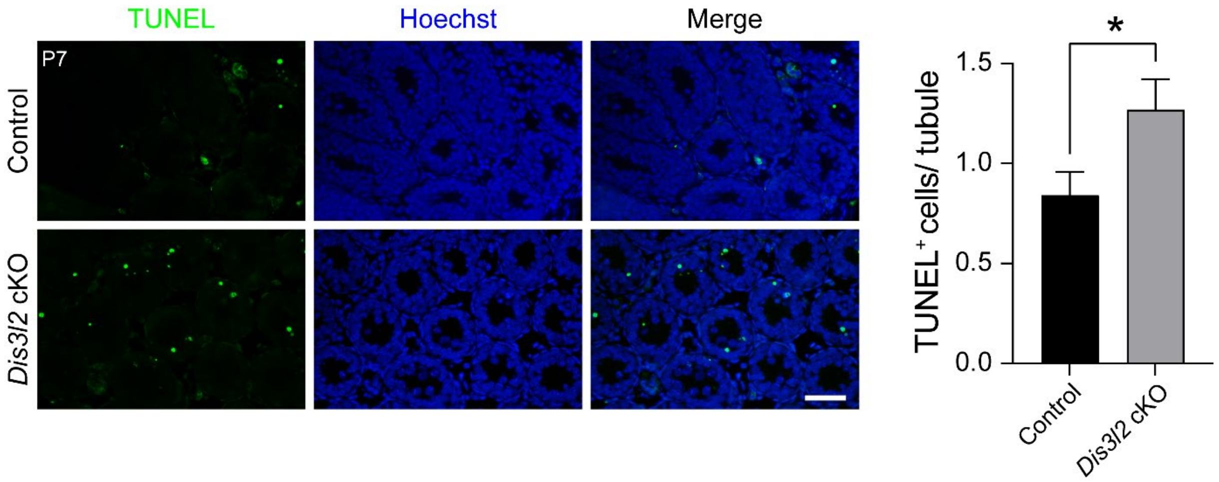

Figure S4. Cell apoptosis in P7 *Dis3l2* cKO testes. Cellular apoptosis was analyzed with TUNEL assay in P7 control and *Dis3l2* cKO testes (left panel). DNA was stained with Hoechst 33342. Scale bar, 50 µm. The number of apoptotic cells per tubule was analyzed from P7 control and *Dis3l2* cKO testes (right panel). Mean ± s.d., n = 3 biologically independent testes from three different mice. *$P < 0.05$.

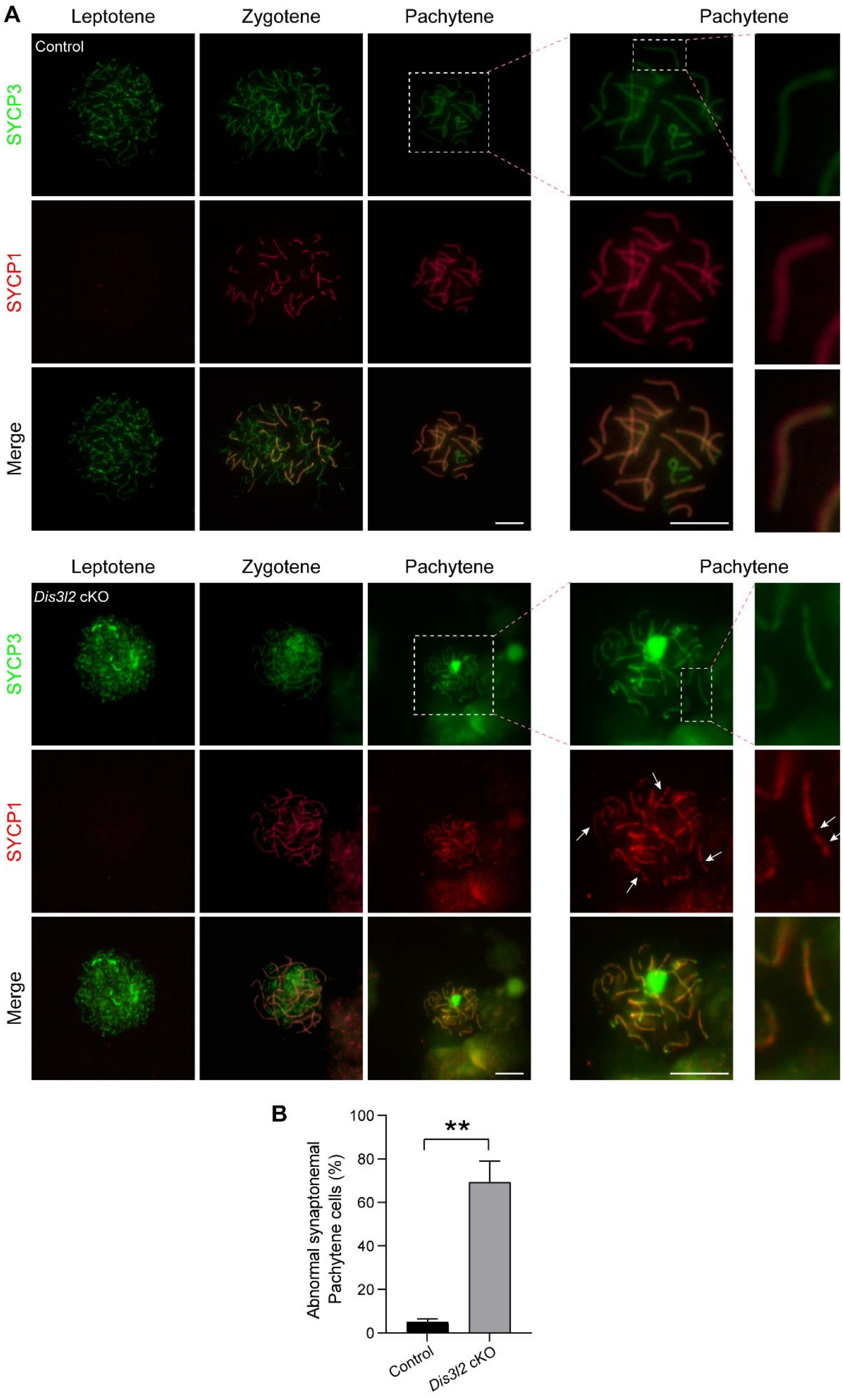

A
Leptotene
Zygotene
Pachytene
Pachytene
Control
SYCP3
SYCP1
Merge
Leptotene
Zygotene
Pachytene
Pachytene
Dis3l2 cKO
SYCP3
SYCP1
Merge
B
Abnormal synaptonemal Pachytene cells (%)
100
80
60
40
20
0
**
Control
Dis3l2 cKO

Figure S5. Abnormal chromosome synapsis in P13 *Dis3l2* cKO testes. (A) Chromosome spreads of spermatocytes from P13 control and *Dis3l2* cKO mice. Co-staining was performed with antibodies to SYCP3 and SYCP1. Arrowheads indicate discontinued SYCP1 signals on the chromosomes. Scale bar, 10 μm. (B) Statistical analysis of the proportion of abnormal synaptonemal pachytene spermatocytes. Mean ± s.d., n = 3 biologically independent testes from three different mice. ***P* < 0.01.

A

The distribution of different classes of transcripts

Biotype	Genes_up	Genes_down
miRNA	1	1
lincRNA	10	40
snRNA	0	2
snoRNA	0	1
scaRNA	2	0
mt tRNA	0	0
mt rRNA	0	0
rRNA	0	0
IG_C_gene	1	0
TEC	1	3
processed transcript	8	25
antisense	7	40
protein coding	3,227	2,580
bidirectional promoter lncRNA	4	8
processed pseudogene	9	24
transcribed processed pseudogene	0	7
transcribed unprocessed pseudogene	1	7
unprocessed pseudogene	4	4
transcribed unitary pseudogene	1	2
polymorphic pseudogene	1	1
sense overlapping	0	1
Total	3,277	2,746

B

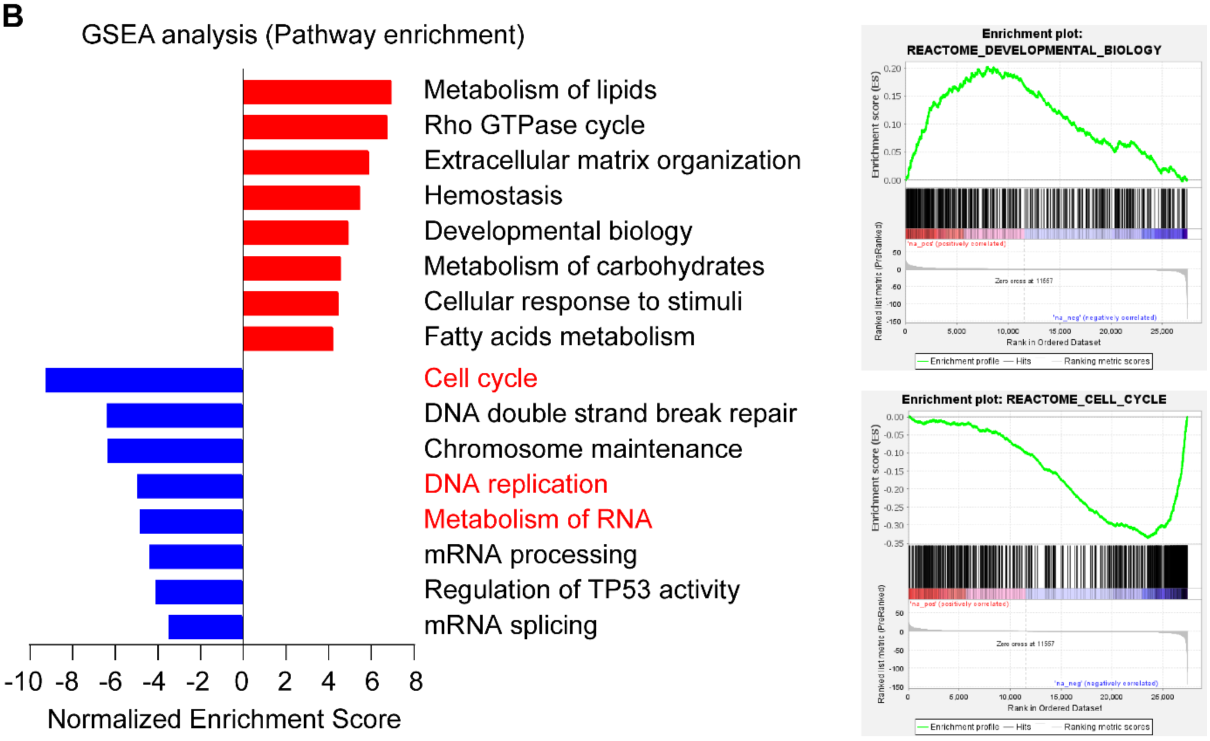

Figure S6. Biotypes of DEGs and pathway enrichment analysis of RNA-seq data. (A) The distribution of genes with upregulated and downregulated expression over different classes of transcripts. (B) Pathway enrichment analysis of *Dis3l2* cKO mouse testes compared with control mouse testes using GSEA software program and the MSigDB C2 curated gene sets (left panel).

Examples of the enrichment plot for terms of developmental biology and cell cycle (right panel).

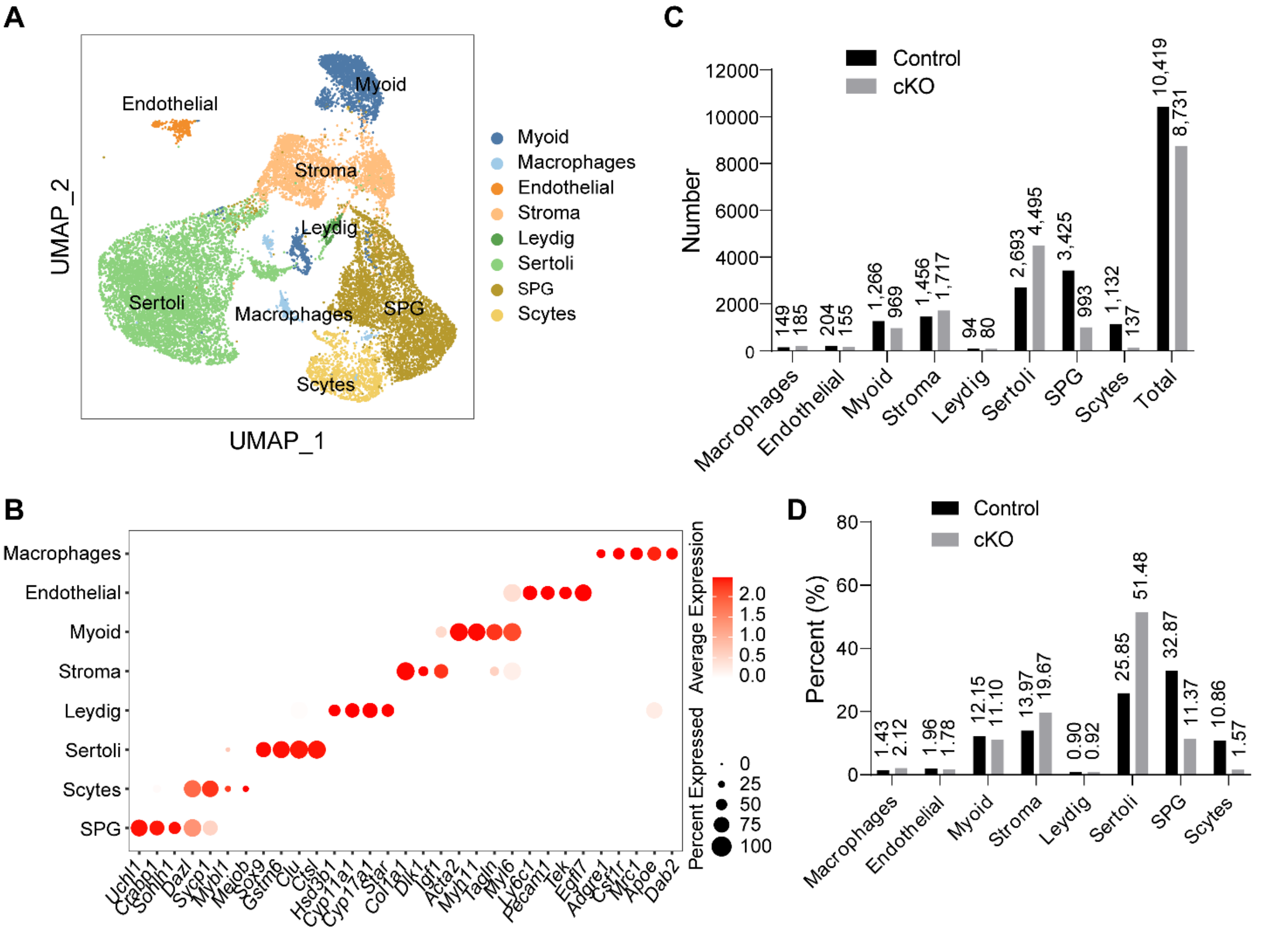

Figure S7. scRNA-seq analysis of P15 control and *Dis3l2* cKO testes. (A) UMAP plot of identified cell clusters from P15 control and *Dis3l2* cKO testicular cells. Each dot represents a single cell and cell types are indicated by colors. (B) Dot plot for the expression of selected marker genes across all identified cell types. (C) The exact cell number of each cell type in control and *Dis3l2* cKO testes. (D) The percentage of each cell type in control and *Dis3l2* cKO testes.

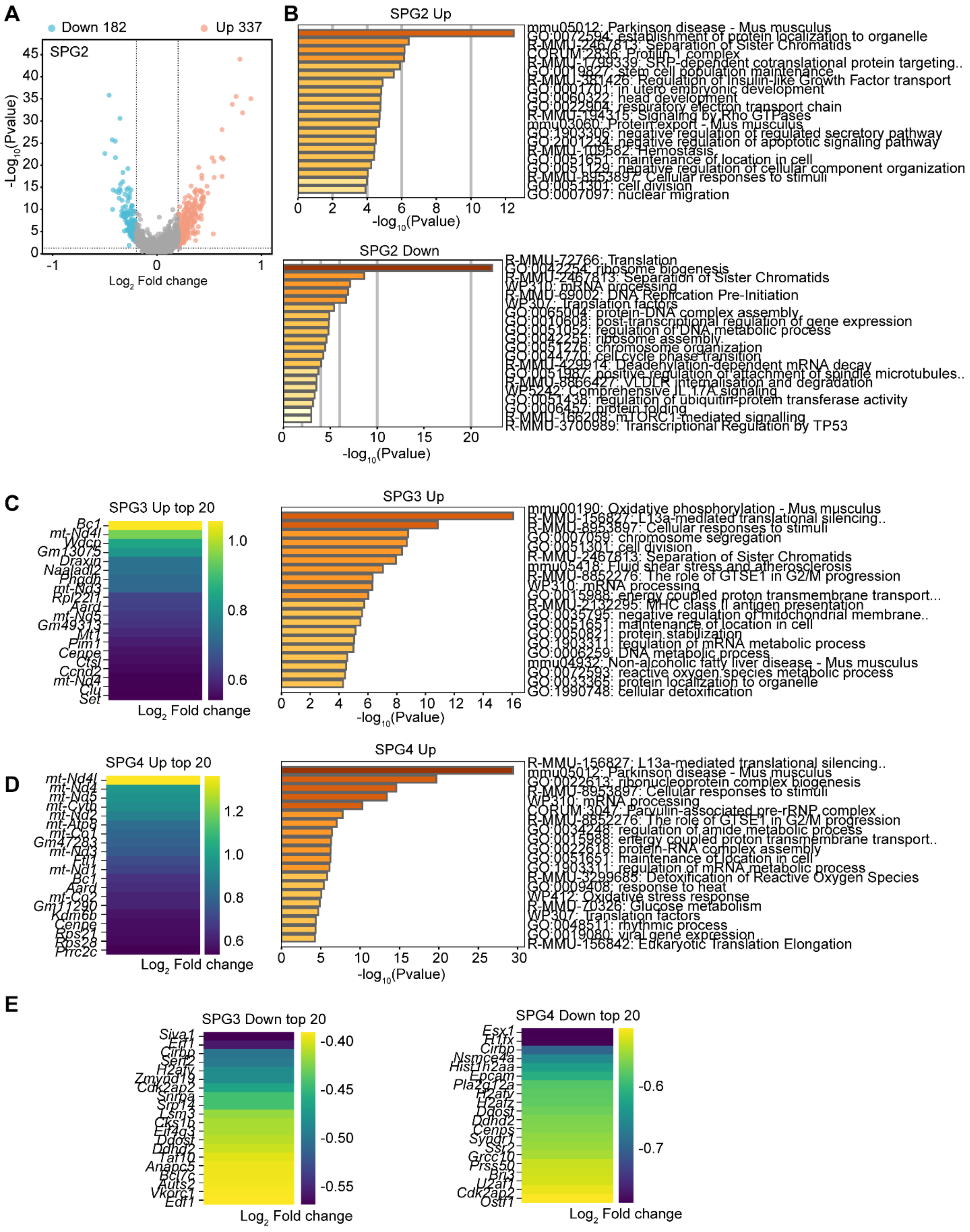

Figure S8. Differentially expressed genes and GO analysis of SPG2, SPG3, and SPG4 cells.

(A) Volcano plot of DEGs in *Dis3l2* cKO SPG2 cells using a cutoff of $P < 0.05$ and $\log_2$ fold-change of > 0.2. (B) GO analysis of upregulated and downregulated transcripts in the SPG2 subtype using Metascape. (C) Top 20 transcripts of upregulated DEGs in *Dis3l2* cKO SPG3 cells (left panel) and enriched GO terms and pathways of the upregulated genes (right panel) using Metascape in *Dis3l2* cKO SPG3 cells. (D) Same as (C), but from *Dis3l2* cKO SPG4 cells. (E) Top 20 downregulated genes in *Dis3l2* cKO SPG3 (left panel) and SPG4 (right panel) cells.

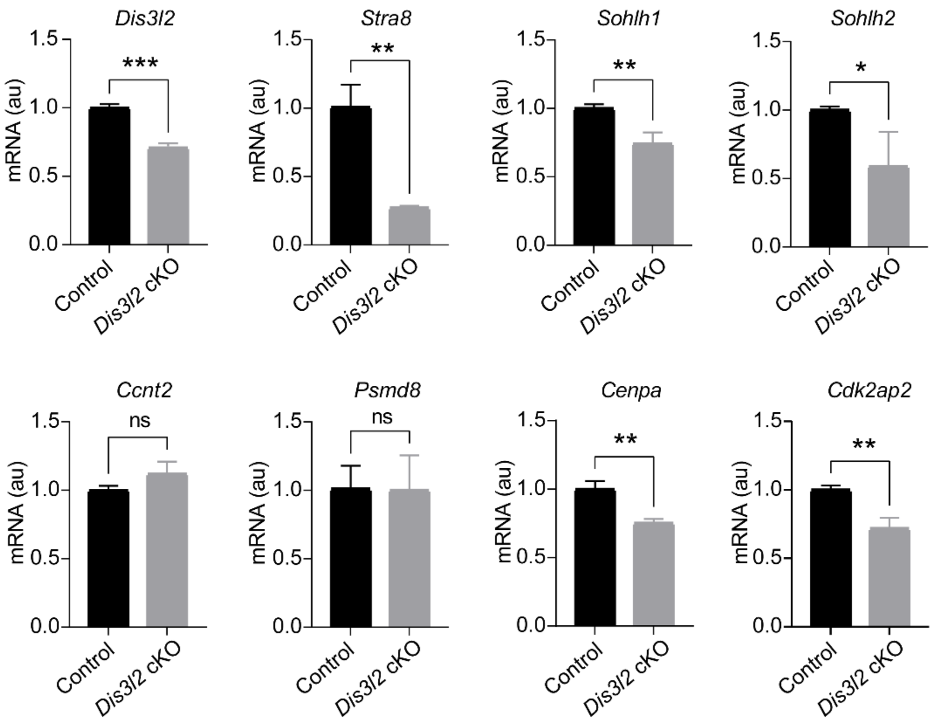

Figure S9. The expression levels of transcripts associated with spermatogonial differentiation and cell cycle. Quantitative RT-PCR analysis of several genes involved in spermatogonial differentiation (up panel) and cell cycle (bottom panel) in P8 control and *Dis3l2* cKO testes. For comparison, the abundance (relative to *β-actin*) of each gene in control testes was set to 1. Data are presented as mean ± s.d for n=3 biologically independent samples per condition.

ns, no significance, $^{*}P < 0.05$, $^{**}P < 0.01$, and $^{***}P < 0.001$.

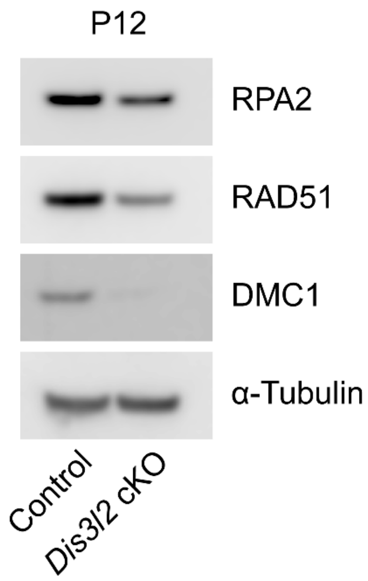

Figure S10. The expression levels of proteins associated with meiosis. Immunoblot assay of RPA2, RAD51 and DMC1 in P12 control and *Dis3l2* cKO testes using α-Tubulin as a load control. Representative of n = 3 biologically independent replicates with similar results per condition.

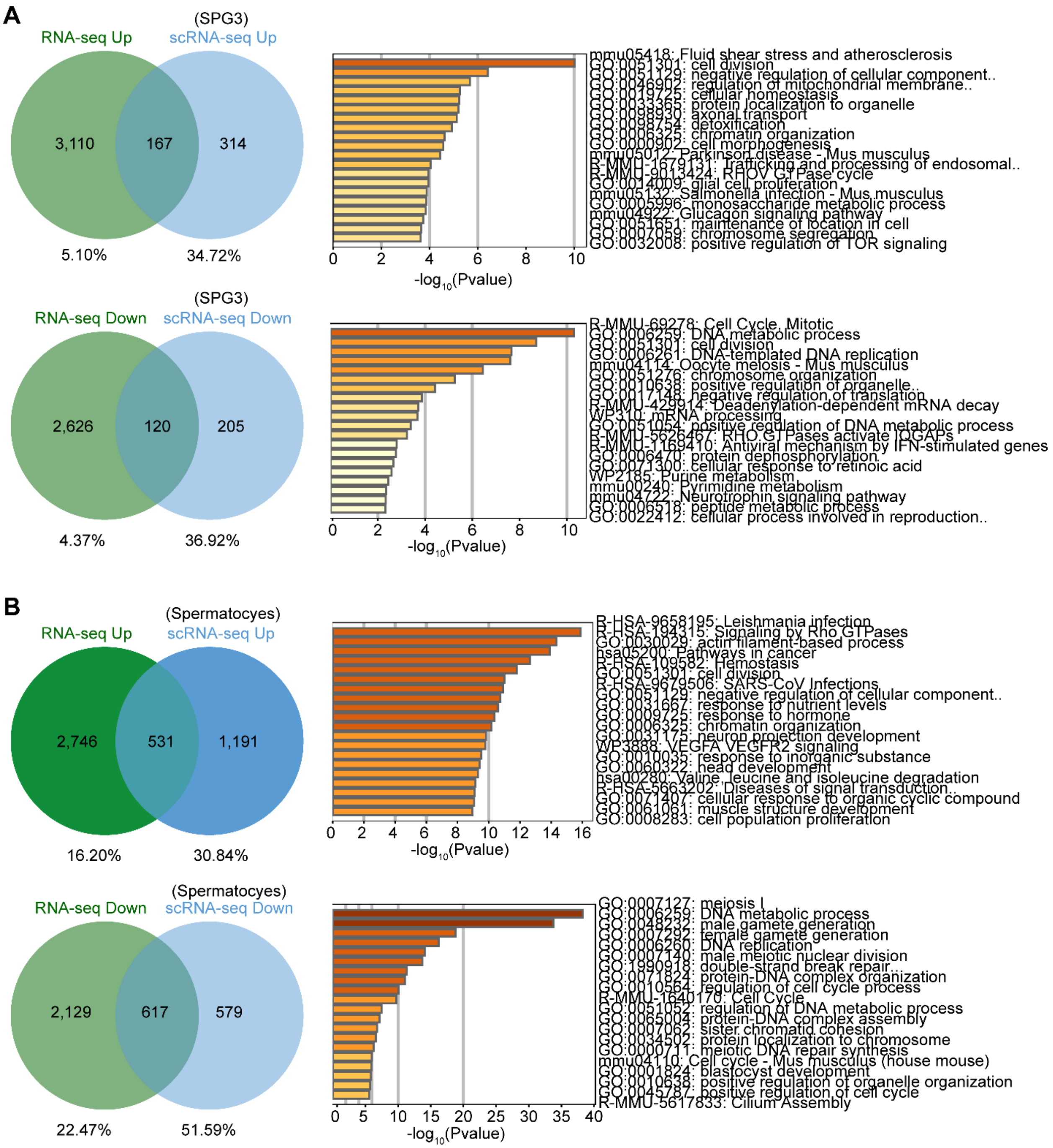

Figure S11. Comparison of bulk RNA-seq and scRNA-seq data. (A) Venn diagrams showing the overlap and difference in the number of upregulated and downregulated transcripts between the bulk RNA-seq data from testes and the scRNA-seq data from SPG3 cells (left panel). GO analysis of the upregulated and downregulated transcripts in both sequencing datasets (right panel)

using Metascape. (B) Same as (A), but showing the results between the bulk RNA-seq data from testes and the scRNA-seq data from spermatocytes.

Table S1. Genotyping primers for *Dis3l2* cKO and *Stra8-Cre* mice

Gene	Direction	Primer (5'-3')
Dis3l2 Flox	F[1]	TTTCCTTCGTAGCAGTATTGGG
	R	TCACATATCATAAAGCCCTACCATC
Dis3l2 Deletion	F	TTTCCTTCGTAGCAGTATTGGG
	R	ATCTTAACATCACTGTCCCCAAGAA
Stra8-Cre (Control)	F	ACTCCAAGCACTGGGCAGAA
	R	GCCACCATAGCAGCATCAAA
Stra8-Cre	F	ACTCCAAGCACTGGGCAGAA
	R	CGTTTACGTCGCCGTCCAG

[1]F, forward; R, reverse

Table S2. Antibodies used in this study

Antibody	Company	Identifier	Immunohisto-chemistry	Immuno blot
Mouse anti-DDX4	Abcam	Cat# ab27591; RRID: AB_11139638	1:200	
Rabbit anti-DDX4	Abcam	Cat# ab13840; RRID: AB_443012	1:200	
Rabbit anti-WT1	Abcam	Cat# ab89901; RRID: AB_2043201	1:200	
Mouse anti-SYCP3	Abcam	Cat# ab97672 RRID: AB_10678841	1:200	
Goat anti-PLZF	R&D Systems	Cat# AF2944; RRID: AB_2218943	1:200	
Rabbit anti-DIS3L2	Novus	Cat# NBP2-38264		1:1000
Rabbit anti-RPA2	Abcam	Cat# Ab76420		1:1000
Rabbit anti-Cyclin D1	Abcam	Cat# ab134175; RRID: AB_2750906	1:200	
Rabbit anti-RAD51	Thermo Fisher Scientific	Cat# PA5-27195		1:1000
Rabbit anti-DMC1	Proteintech	Cat# 13714-1-AP		1:1000
Rabbit anti-Actin	ABclonal	Cat# AC026		1:10000
Mouse anti-alpha tubulin	Thermo Fisher	Cat# 62204;		1:1000

	Scientific	RRID: AB_1965960		
Goat anti-KIT	R&D Systems	Cat# AF1356;<br>RRID: AB_354750	1:200	
Rabbit anti-phospho-histone H2A.X (Ser139)	Cell Signaling Technology	Cat# 9718;<br>RRID: AB_2118009	1:200	
Donkey anti-mouse IgG, Alexa Fluor 488	Thermo Fisher Scientific	Cat# A-21202	1:200	
Donkey anti-rabbit IgG, Alexa Fluor 594	Thermo Fisher Scientific	Cat# A-21207	1:200	
Donkey anti-goat IgG, Alexa Fluor 488	Thermo Fisher Scientific	Cat# A-11055	1:200	
Donkey anti-mouse IgG, Alexa Fluor 594	Thermo Fisher Scientific	Cat# A-21203	1:200	
Goat anti-mouse IgG, HRP	Thermo Fisher Scientific	Cat# 62-6520		1:5000
Goat anti-rabbit IgG, HRP	Thermo Fisher Scientific	Cat# 31460		1:5000

Table S3. Primers used for RT-PCR

Gene	Direction	Primer (5’-3’)
Dis3l2	F[1]	AGGAGGATGTTTCAGAAGGCT
	R	GGCTCTATTACGAGCAACAACT
Plzf	F	CTGGGACTTTGTGCGATGTG
	R	CGGTGGAAGAGGATCTCAAACA
Dazl	F	ATGTCTGCCACAACTTCTGAG
	R	CTGATTTCGGTTTCATCCATCCT
Pramef12	F	TACAGCTCGCAATGCAAAGC
	R	CCTCAGGATGTTAAGTCGTTTGT
Cpeb1	F	GGACTCCCGTTCTAGCAGC
	R	GGAAATGCGAAGGCTTGAAATCA
Spo11	F	CGTGGCCTCTAGTTCTGAGGT
	R	GCTCGATCTGTTGTCTATTGTGA
Rhox10	F	GCCCAATGTCGCTTTGGAAG
	R	AGCTGTAGGTTTGCGTGTATTT
Sohlh2	F	GGGCAGGGCAGAGTAAATCTT
	R	CAAACGAGTTAGCAGCCAAAAG
Sohlh1	F	CGGGCCAATGAGGATTACAGA
	R	TCCTGCGTTCTCTCTCGCT
Stra8	F	CAAAAGCCTTGGCTGTGTTA
	R	AAAGGTCTCCAGGCACTTCA
Sycp1	F	CAAAAGCCCTTCACACTGTTCG

	R	GTTTTCCCGACTGGACATTGTAA
Sycp2	F	AGGATGAGATCACTACACCTAGC
	R	GGTGACGCAGCATAATCCATT
Sycp3	F	AGCCAGTAACCAGAAAATTGAGC
	R	CCACTGCTGCAACACATTCATA
Syce1	F	GCATGTTGCAGGAGTGTAAAGA
	R	GCTGCTGTCCAAAACACACATC
Syce2	F	TGGACTCTAGCATTGAAACCCT
	R	TCCTGAATGATTTTGCTGTGGT
Rec8	F	TATGTGCTGGTAAGAGTGCAAC
	R	TGTCTTCCACAAGGTACTGGC
Mael	F	CCTCCCTTGTGAAATTGGCTG
	R	AATGGAATCGAAATCCTCGTGG
Dmc1	F	CCCTCTGTGTGACAGCTCAAC
	R	GGTCAGCAATGTCCCGAAG
Rad51	F	AAGTTTTGGTCCACAGCCTATTT
	R	CGGTGCATAAGCAACAGCC
Hormad1	F	GGCTCCTAGCTGTTTCAGTATCT
	R	TTGTCCCATAAGCACGTTCTG
Piwil1	F	AGACCTCATTGGAAGGTGTCA
	R	TGTTCCCCATTCCGAGTCTGA
Piwil2	F	TTGGCCTCAAGCTCCTAGAC
	R	GAACATGGACACCAAACCTACA

Tdrd9	F	TACGTGCTAGACCACTACACC
	R	AACGCTCCTTACTGATCCACC
Ccnt2	F	AGCAAGCAAGGATTTGGCAC
	R	CACATGCTATCACTGTGGGTTT
Psmd8	F	GGCATGTACGAGCAACTCAAG
	R	GCTCAAGCAGAACCAACTTCA
Cenpa	F	CTCCAGTGTAGGCTCTCAGAC
	R	CTGAAAGGCTTCTTCCTGAACA
Cdk2ap2	F	CTGTTTAACGACTTTGGTCCGC
	R	CAGACAGCAGGTCCGTGTAG
β-actin	F	GGCTGTATTCCCCTCCATCG
	R	CCAGTTGGTAACAATGCCATGT

[1]F, forward; R, reverse
